# The First Prokaryotic Trehalose Synthase Complex Identified in the Hyperthermophilic Crenarchaeon *Thermoproteus tenax*

**DOI:** 10.1371/journal.pone.0061354

**Published:** 2013-04-23

**Authors:** Melanie Zaparty, Anna Hagemann, Christopher Bräsen, Reinhard Hensel, Andrei N. Lupas, Henner Brinkmann, Bettina Siebers

**Affiliations:** 1 Institute for Molecular and Cellular Anatomy, University of Regensburg, Regensburg, Germany; 2 Department of Chemistry, Biofilm Centre, Molecular Enzyme Technology and Biochemistry, University of Duisburg-Essen, Essen, Germany; 3 Department of Biology, Microbiology I, University of Duisburg-Essen, Essen, Germany; 4 Department of Protein Evolution, Max-Planck-Institute for Developmental Biology, Tübingen, Germany; 5 Département de Biochimie, Faculté de Médecine, Université de Montréal, Montreal, Canada; Instituto de Biología Molecular y Celular de Plantas, Spain

## Abstract

The role of the disaccharide trehalose, its biosynthesis pathways and their regulation in Archaea are still ambiguous. In *Thermoproteus tenax* a fused trehalose-6-phosphate synthase/phosphatase (TPSP), consisting of an N-terminal trehalose-6-phosphate synthase (TPS) and a C-terminal trehalose-6-phosphate phosphatase (TPP) domain, was identified. The *tpsp* gene is organized in an operon with a putative glycosyltransferase (GT) and a putative mechanosensitive channel (MSC). The *T. tenax* TPSP exhibits high phosphatase activity, but requires activation by the co-expressed GT for bifunctional synthase-phosphatase activity. The GT mediated activation of TPS activity relies on the fusion of both, TPS and TPP domain, in the TPSP enzyme. Activation is mediated by complex-formation *in vivo* as indicated by yeast two-hybrid and crude extract analysis. In combination with first evidence for MSC activity the results suggest a sophisticated stress response involving TPSP, GT and MSC in *T. tenax* and probably in other Thermoproteales species. The monophyletic prokaryotic TPSP proteins likely originated via a single fusion event in the Bacteroidetes with subsequent horizontal gene transfers to other Bacteria and Archaea. Furthermore, evidence for the origin of eukaryotic TPSP fusions via HGT from prokaryotes and therefore a monophyletic origin of eukaryotic and prokaryotic fused TPSPs is presented. This is the first report of a prokaryotic, archaeal trehalose synthase complex exhibiting a much more simple composition than the eukaryotic complex described in yeast. Thus, complex formation and a complex-associated regulatory potential might represent a more general feature of trehalose synthesizing proteins.

## Introduction

The necessity of adaptation to changing environmental conditions like temperature and salinity is most severe for organisms thriving in extreme habitats, such as (hyper)-thermophiles or extreme halophiles. Beside intrinsic factors, i.e. structural/mechanistic adaptation of macromolecules such as proteins or membranes, also extrinsic factors like osmo-, thermo- or cryoprotectants are intracellularly accumulated to stabilize cellular constituents and to respond to various stress conditions. These compounds, referred to as compatible solutes because they do not interfere with metabolism, include polyols (e.g. mannitol or sorbitol), amino acids (glutamic acid and proline), quarternary ammonium salts (i.e. glycine betaine), and disaccharides, e.g. trehalose and sucrose [1].

Trehalose (α-D-glucopyranosyl-1,1-α-D-glucopyranoside), a non-reducing disaccharide, is distributed in all three domains of life. The sugar gained special interest due to its multifunctional properties as carbon and energy source as well as its predominant role as compatible solute in response to various stresses including temperature (heat, cold), oxidation, osmolarity, dehydration or desiccation [2]–[10].

Prokaryotes utilize multiple pathways for trehalose biosynthesis [11]–[13] including the trehalose-6-phosphate synthase (TPS)/trehalose-6-phosphate phosphatase (TPP) pathway (TPS/P pathway) (for archaeal trehalose biosynthesis pathways see Figure S1 in File S1), whereas Eukaryotes exclusively utilize the TPS/P pathway, which thus represents the ubiquitous and most commonly used pathway for trehalose synthesis [11], [12].

This pathway comprises two enzymes: Trehalose-6-phosphate synthase (TPS; OtsA in *E. coli*; EC 2.4.1.15) catalyzes the transfer of glucose from UDP-glucose (UDPG) to glucose-6-phosphate (G6P), forming trehalose-6-phosphate (T6P) and UDP. Trehalose-6-phosphate phosphatase (TPP; OtsB in *E. coli*, EC 3.1.3.12) dephosphorylates T6P, yielding trehalose and P_i_ (Figure S1 in File S1) [14], [15].

In bacterial genomes TPS and TPP enzymes are encoded by single genes and usually clustered in operon-like structures, whereas in Eukaryotes both, *tps* and *tpp* genes, are mostly fused to *tpsp* genes encoding TPSP fusion proteins which exhibit either only TPS or TPP activity or in the case of plant class II enzymes are catalytically inactive [16]. In Bacteria, such TPSP fusions have only been identified in few species and bifunctional activity of a TPSP fusion protein has only been demonstrated for the bacterium *Cytophaga hutchinsonii* [16]. In Eukaryotes, the TPS/P pathway has been studied most extensively in yeast, where trehalose is synthesized via a complex comprising four proteins [17]–[20]. Complex formation was discussed to have regulatory implications [17]. However, a trehalose synthesizing protein complex has never been observed in Prokaryotes so far.

In the hyperthermophilic Crenarchaeon *T. tenax* trehalose has previously been identified as the sole compatible solute [3]. Here, we report a detailed study of the TPS/P pathway in this organism involving a TPSP fusion enzyme with bifunctional activity. The *tpsp* gene forms an operon with two ORFs encoding a glycosyltransferase (GT) and a mechanosensitive channel (MSC), respectively. Enzymatic studies revealed that the *T. tenax* TPSP only possesses phosphatase (TPP) activity and requires activation by complex formation with the GT for full, bifunctional TPSP activity. This is the first report of a prokaryotic trehalose synthase complex and a general model of stress response in *T. tenax* involving all three proteins encoded by the *msc-gt-tpsp* operon is proposed.

## Materials and Methods

### Strains and growth conditions

Mass cultures of *T. tenax* Kra1 (DSM 2078) [21] were grown under autotrophic conditions as described previously [22] in a medium described by Brock et al. [23] with slight modifications. *Escherichia coli* strains DH5α (Life Technologies), BL21-CodonPlus(DE3)-RIL (Agilent Technologies), Rosetta(DE3) (Life Technologies) and Lemo21(DE3) (NEB) for cloning and expression studies were cultured under standard conditions following the instructions of the manufacturer.

### Cloning and heterologous expression in *E. coli*

Genomic DNA of *T. tenax* was prepared by using DNAzol reagent according to the instructions of the manufacturer (Life Technologies). The genes TTX_1304 (TPSP), TTX_1305 (GT), the TPS coding region of TTX_1304 (position 39–1,311), the TPP coding region of TTX_1304 (position 1438–2196 of the *tpsp* gene) and TTX_1304a (MSC) were cloned via PCR mutagenesis with genomic DNA as template using *Pfu* (Fermentas) or KOD polymerase (Merck Bioscience). The following vectors, primer sets and restriction site (underlined) were used: TTX_1304 (pET24a, forward primer TTCCGTGGGAGGACATATGCG, reverse primer CGCCAGCGGCGAATTCTAGAGACAGGGG, *Nde*I/*Eco*RI), TTX_1305 (pET15b, forward primer TCCTCAACATATGAACGTAGC, reverse primer CTCCCACGGATCCCCTTTTTAC, *Nde*I/*Bam*HI), *tpp* coding region of TTX_1304 (pET15b, forward primer GAGAAGGCCCTCAGACATATGGA, reverse primer GGTTGAATTCTTAGCCCGCGG, *Nde*I/*Eco*Ri), *tps* coding region of TTX_1304 (pET24a, forward primer GGCCGGGAATTCGTGCGCCTCATAGTGGTC, reverse primer CCGGCCGTCGACTTAGATTAGGGAGTAGATGAAG, *Eco*RI/*Sal*I) and TTX_1304a (pET24a, forward primer ATATTCGCGCGGCGGGCCCCATATGGGACT, reverse primer CCAACGGGAATTCTGCGGCGC, *Nde*I/*Eco*RI). Successful cloning was confirmed by sequencing (LGC Genomics, Berlin). Heterologous expression was carried out in *E. coli* BL21-CodonPlus(DE3)-RIL (*tpp* coding region), Rosetta (DE3) (*tpsp*) or Lemo21(DE3) (*gt* and *tps*) using the pET expression system (Novagen). Cells were grown in LB medium containing the appropriate antibiotics at 37°C and expression was induced by addition of 1 mM isopropyl-1-thio-β-D-galactopyranoside (IPTG), after induction cells were grown for further 4 h. Lemo21(DE3) cells were grown with additional 2 mM L-rhamnose, induced with 0.4 mM IPTG and expression was carried out overnight at 22°C. After expression cells were harvested by centrifugation (6000×g, 4°C, 15 min).

### Purification of recombinant enzymes

*E. coli* cells were suspended (3 ml/1 g cells) in buffer A (100 mM Tris/HCl, pH 7.0 (70°C) containing 7.5 mM DTT) (TPSP, TPP) or LEW buffer (Machery-Nagel, Düren, Germany) (GT, TPS) and passed three times through a French pressure cell at 150 MPa. Cell debris and unbroken cells were removed by centrifugation (60,000×*g* for 30 min at 4°C). The resulting crude extracts were diluted 1∶1 with buffer A or LEW buffer. Heat precipitation was performed at 80°C (isolated TPP and TPS domain and GT) or 90°C (TPSP) for 20 min. After centrifugation at 20,000×*g*, for 30 min at 4°C supernatants containing TPSP or TPP were dialyzed overnight against buffer A containing 5 mM MgCl_2_ at 4°C, GT and TPS containing samples were directly applied to Ni-TED (nickel (tris(carboxymethyl)ethylene diamine)) IMAC (immobilized metal ion affinity chromatography) column (Machery-Nagel, Germany) without prior dialysis.

TPSP and TPP domain were further purified by anion exchange and size exclusion chromatography. Dialyzed probes were applied to a 17 ml Q sepharose column (GE healthcare) equilibrated with 20 mM Tris-HCl pH 7.0 (80°C) containing 5 mM MgCl_2_ and 5 mM DTT and eluted with a linear gradient from 0 to 1 M NaCl in the same buffer. Fractions containing the recombinant enzymes were pooled and concentrated (Vivaspin6, Sartorius Stedim Biotech). After dialysis overnight against 50 mM Tris-HCl pH 7.0 (80°C) containing 5 mM MgCl_2_, 5 mM DTT and 300 mM NaCl, samples were applied to size exclusion chromatography column (HiLoad 26/60 Superdex 200 prep grade, GE healthcare) equilibrated in the same buffer and proteins were eluted with an isocratic flow. The fractions containing the respective recombinant enzymes were analyzed via SDS-PAGE and enzyme activity tests, pooled and further used for the enzymatic measurements.

GT and TPS containing samples were applied to a Ni-TED IMAC gravity flow column (Machery-Nagel, Germany) equilibrated with LEW buffer. Proteins were eluted with elution buffer according to the manufacturer's instructions. The GT or TPS containing fractions were concentrated and subjected to size exclusion chromatography as described above.

### Enzymatic assays

TPSP and TPP activity of purified proteins was qualitatively monitored by detecting the biosynthesis of trehalose from UDPG and G6P as well as from T6P by thin layer chromatography (silica gel G60 plates, Merck) as described previously [24]. The specific TPSP and TPP activities were determined as P_i_ release from UDPG and G6P (i.e. TPSP activity) as well as from T6P (i.e. TPP activity) using the colorimetric SensoLyte malachite green phosphate assay kit (AnaSpec). In both cases a discontinuous assay system was used. Purified TPSP (1 µg) and artificial TPP domain (1 µg) were incubated in the presence or absence of GT (1 µg) at 80°C in 100 mM Tris-HCl (pH 7.0 at 80°C) containing 4 mM MgCl_2_, 10 mM DTT (only when P_i_ was detected), and either 4 mM UDPG and 8 mM G6P (TPSP activity) or 8 mM T6P (TPP activity) in a final volume of 300 µl. After preincubation at 80°C for 3 min the reactions were started by the addition of proteins. Measurements in crude extracts were carried out at 86°C with 50 µg of total protein (cell free extract) in the assay mixture described above.

#### Determination of trehalose via TLC

After 30 min of incubation, the reactions were stopped by the addition of acetone (1∶1) and subsequent incubation at −20°C for 20 min followed by centrifugation (10,000×*g*, 4°C, 20 min). Thin layer chromatography of the supernatant was carried out according to [24] on silica gel G60 plates (Merck) with butanol∶ethanol∶water (v/v/v 5∶3∶2) as solvent at room temperature.

#### Determination of P_i_ via malachite green assay

Every minute (up to 5 min) samples were taken (40 µl), diluted 1∶1 with 100 mM Tris-HCl (pH 7.0 at 80°C), stopped by the addition of 20 µl malachite green reagent (according to the manufacturer's instructions (AnaSpec)) and transferred to a 96 well plate. The samples were shaken for 5 min in the plate reader (Tecan), the extinction of each sample was determined at 620 nm and the P_i_ amount formed was calculated using a phosphate standard calibration curve following the manufacturer's instructions. From the time depended P_i_ increase the specific activities were calculated.

#### Determination of UDP via spectrophotometric assay

The specific TPS activities were determined as UDP release from UDPG and G6P by coupling the formation of UDP to the oxidation of NADH via pyruvate kinase (rabbit muscle, EC 2.7.1.40, Sigma) and L-lactate dehydrogenase (rabbit muscle, EC 1.1.1.27, Sigma) in a discontinuous assay system. Partially purified TPSP (25 µg) and artificial TPS domain (25 µg) were incubated in the presence or absence of partially purified GT (25 µg) at 80°C in 100 mM Tris-HCl (pH 7 at 80°C) containing 20 mM MgCl_2_, 10 mM DTT and 8 mM G6P in a final volume of 1000 µl. After preincubation at 80°C for 5 min the reactions were started by the addition of 4 mM UDPG. At the time points indicated 100 µl samples were taken, stopped on ice and added to 125 mM Tris-HCl (pH 7.5, RT) containing 20 mM MgCl_2_, 2 mM phosphoenolpyruvate, 0.1 mM NADH, 8 units of pyruvate kinase, and 4 units of L-lactate dehydrogenase in a final volume of 500 µl for quantitative UDP determination. After 15 min incubation at 37°C decrease in absorption was determined at 340 nm (εNADH, RT = 6.2 mM^−1^cm^−1^). Negative controls without TPSP, TPS or GT, respectively and without UDP-glucose or G6P, as well as tests with the artificial TPP domain were performed.

### Measurements in crude extracts of *T. tenax*

Crude extracts of *T. tenax* cells grown on glucose were prepared by resuspension of 1 g cells (wet weight) in 3 ml 100 mM HEPES/KOH, pH 7.0 (86°C) containing 10 mM β-mercaptoethanol and 5 mM MgCl_2_, and subsequent disruption by passing three times through a French pressure cell at 150 MPa. Cell debris was removed by ultracentrifugation (60,000×*g*, 45 min, 4°C). TPSP activity measurements were carried out as described above at 86°C in 100 mM Tris-HCl, pH 7.0 in the presence of 10 mM DTT in a total volume of 125 µl with 50 µg of total protein. Trehalose formation was qualitatively detected using TLC as described above.

### Northern blot analysis

Total RNA was prepared from autotrophically grown *T. tenax* cells by using TRIzol reagent and RNeasy Protect Mini Kit according to the instructions of the manufacturer (Life Technologies, Qiagen). Integrity of the RNA was checked by agarose gel electrophoresis using a 20 mM MOPS/6% formaldehyde buffer system. Concentration and purity was determined spectrophotometrically.

As template for subsequent probe generation, internal gene fragments of the *tps* and *tpp*, coding regions of *tpsp*, were cloned into the *Eco*RI and *Bam*HI restriction sites (underlined) of the vector pSPT19 (Roche Diagnostics) using the primers AGAGGCGGCGGAATTCGGAG (sense) and TACAGAGGGATCCTGCGAGG (antisense) for *tps* fragment (corresponding to nucleotide position 476–748 of the entire *tpsp* gene) as well as CGTTGGTCCCCCAGAATCCC (sense) and TCCGGGGATCCTAGAGGCGA (antisense) for the *tpp* fragment (corresponding to position 1539–1798 of the entire *tpsp* gene). Digoxygenin (DIG)-labelled antisense mRNA was generated by *in vitro* transcription using T7-RNA polymerase and the respective recombinant vector constructs as template.

After capillary RNA transfer to positively charged nylon membranes (Roche Diagnostics), blots were hybridized with the DIG-labeled RNA probes according to the manufacturer's instructions. Hybridization was performed overnight at 68°C in DIG Easy Hyb solution (Roche Diagnostics). Low stringency washes were performed two times for 5 min at RT using low-stringency buffer (2×SSC, 0.1% SDS) following high stringency washes using 0.05–0.1×SSC, 0.1% SDS, at 68°C–70°C for 2×15 min.

Signal detection was carried out with a ChemiDoc detection system (BioRad) and the transcript lengths were determined using a molecular size standard (Fermentas).

### Yeast two-hybrid

Yeast two-hybrid analyses were carried out by using the Matchmaker system 3 (Clontech). Genes encoding TPSP (TTX_1304) and GT (TTX_1305) were amplified from the *T. tenax* genomic DNA by PCR using the following primer sets (underlined sequences are homologous to the vectors): TTX_1304 (forward primer GAGGACCTGCATATGCGCCTCATAGTGGTCTCCAACAG, reverse primer ATGCGGCCGCTGCAGAGGGGGGCGCAACTGCTCCAATAG); TTX_1305 (forward primer GATTACGCTCATATGAACGTAGCTGTAGTGGCGCCGCAG, reverse primer CATCTGCAGCTCGAGCGGTTGTGCCCCTAAAATTAATTC). PCR products were cloned into the pGADT7 and pGBKT7 linearized with *Eco*RI and *Bam*HI using the In-Fusion® cloning system according to the manufacturer's instructions (Clontech). Successful cloning was confirmed by sequencing. Protein–protein interactions were detected by co-transformation of yeast AH109 [25] with pGADT7::*TTX_1305* and pGBKT7::*TTX_1304* according to the manufacturer's instruction. The cells were then plated on selective SD medium lacking leucine and tryptophan and incubated for three days at 30°C to check for transformation with both plasmids. The resulting colonies were cultivated in liquid SD medium lacking leucine and tryptophan, grown to an OD_600_ of 1.5 and diluted to an OD_600_ of 0.1. 5 µl were dropped on SD plates supplemented with X-α-gal but lacking leucine, tryptophan, histidine and adenine to check for interaction of TTX_1305 with TTX_1304 [26].

### Phylogenetic analyses

Homology searches were performed with various query sequences at the non-redundant protein database of NCBI (http://blast.ncbi.nlm.nih.gov/Blast.cgi) using the Blastp option [27]. Sequences were selected in function of their blast score and length using the alipapa program of the Must package [28] and a first alignment was created by Clustal W 1.83 [29], manual refinement was done in the edit program of the Must package. The final datasets were created after the removal with G-blocks of highly variable and ambiguously aligned positions including those with at least 50% gaps [30]. Phylogenetic trees with both, the maximum likelihood and Bayesian methods were inferred with RAxML under a LG+F+Γ4 model [31] and with PhyloBayes [32] under the CATfixC20/C60+Γ4 models, respectively. The site-heterogeneous C20–C60 series of empirical profile mixture models was shown to perform better than the standard site-homogeneous models (with a single instantaneous substitutions matrix for all positions), especially if the data are highly saturated as is invariantly the case in deep-level comparisons [33]. One hundred bootstrap replicates using the fast bootstrap option of RAxML [31] and the same model as above were performed.

## Results

### Genome organization of the genes involved in the *T. tenax* TPS/P pathway

**Figure 1:**
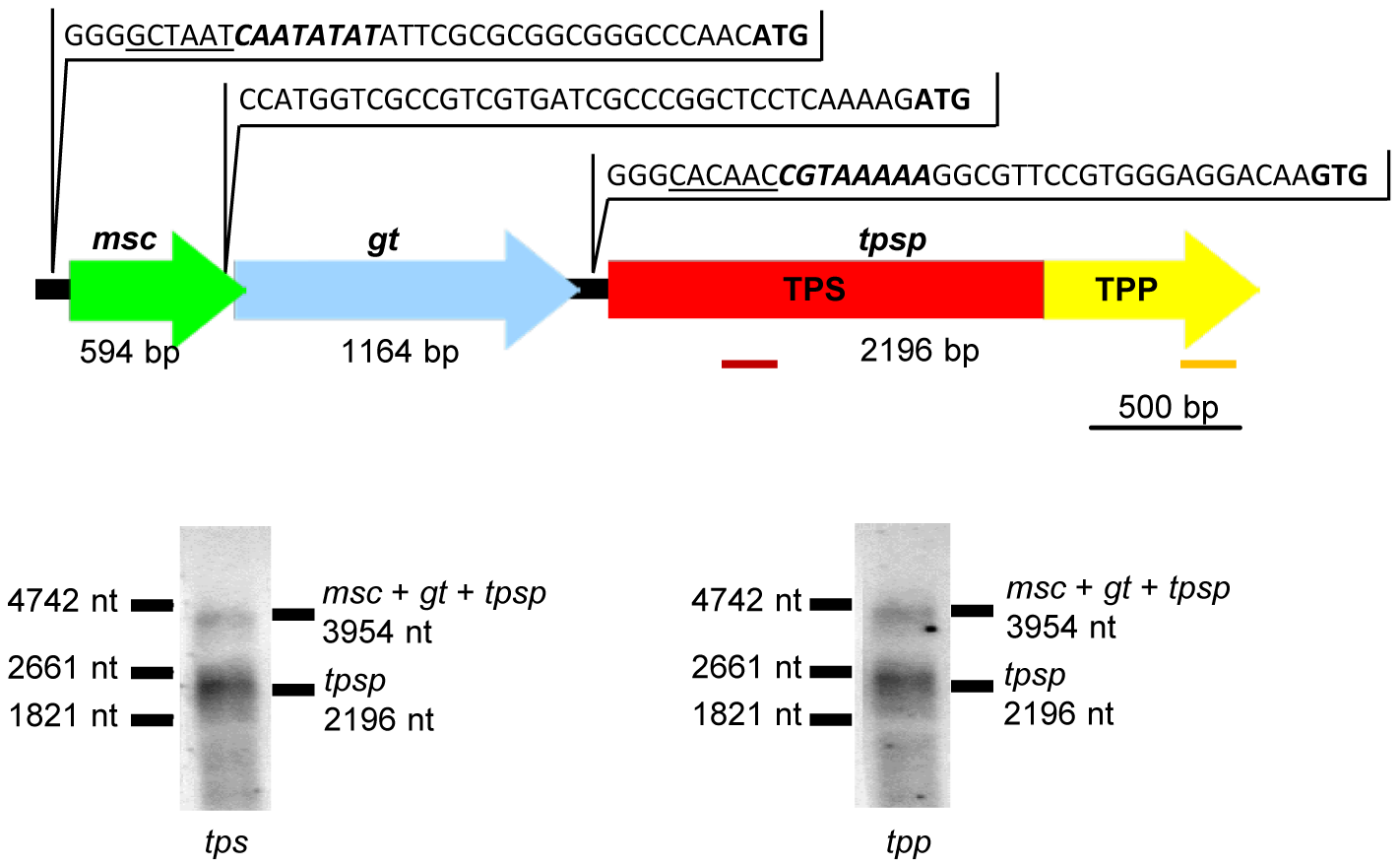
Operon organization and Northern blot analysis of the *tpsp* gene. **Upper panel:** Operon organization of the genes encoding trehalose-6-phosphate synthase/phosphatase (*tpsp*) (red, trehalose-6-phosphate synthase coding region; yellow, trehalose-6-phosphate phosphatase coding region), putative glycosyltransferase (*gt*, blue) and the putative mechanosensitive channel (*msc*, green). Genes are represented by arrows with the respective gene length indicated. Upstream regions of *msc*, *gt* and *tpsp* genes are shown (BRE site (underlined), TATA box (bold and italic) and start codon (bold)). mRNA probes used for Northern Blot analyses are indicated as colored bars below the TPS and TPP encoding regions of the *tpsp* gene. **Lower panel:** Northern Blot analysis of total RNA from *T. tenax* using specific DIG-labeled mRNA probes complementary to trehalose-6-phosphate synthase (*tps*) (left panel) and trehalose-6-phosphate phosphatase (*tpp*) (right panel) coding regions of *tpsp* mRNA, respectively. Total RNA was purified from autotrophically grown *T. tenax* cells and 5–10 µg of RNA were separated by denaturing 1.2% agarose-MOPS/formaldehyde gels [50] and subsequently analyzed. The marker sizes are indicated at the left side of each blot, the expected mono- and tricistronic transcript sizes are given on the right.

In the genome of *T. tenax*, a gene was identified (*tpsp*; TTX_1304), which encodes the trehalose-6-phosphate synthase/phosphatase (TPSP) [34]. This protein represents a fusion of an N-terminal TPS and a C-terminal TPP domain (Figure 1), with the TPS domain belonging to the glycosyltransferase 20 family (CAZy database, http://www.cazy.org/).

Northern blot analyses revealed that the *tpsp* gene forms an operon with two upstream ORFs, TTX_1305 and TTX_1304a (Figure 1). Mono- (*tpsp*) and tricistronic (*msc-gt-tpsp*) transcripts were detected using *tps* and *tpp* domain-specific antisense RNA probes, whereas no single domain *tps*- or *tpp*- transcripts could be identified. This is in accordance with sequence analyses, which identified complete promoter sequences including TATA box, BRE element and start codon only in front of the *msc* and *tpsp* genes, but not in front of the *gt* gene (Figure 1). This genomic organization suggests a functional relationship of the *tpsp* gene with these two ORFs.

Bioinformatic analyses suggested that TTX_1305 (1,164 bp; 387 aa) most likely encodes a GTB-like glycosyltransferase (GT) of the glycosyltransferase 4 family with the closest match to the phosphatidylinositol mannosyltransferase (PimA) from *Mycobacterium smegmatis*. TTX_1304a (594 bp, 197 aa) shows significant structural and sequence similarities to the transmembrane region of the small conductance mechanosensitive channel (MSC) from *E. coli* (HHpred server [35]).

### Cloning, heterologous expression in *E. coli* and purification of the *T. tenax* TPSP, GT, MSC and of the isolated TPS and TPP domain

The *tpsp* gene (2,196 bp), the *gt* gene (1,164 bp) and a truncated 759 bp fragment encoding the C-terminal TPP domain (position 1,438–2,196 of the *tpsp* gene), as well as truncated 1,272 bp fragment encoding the N-terminal TPS domain (position 39–1,311 of the *tpsp* gene) of *T. tenax* TPSP were cloned and expressed in *E. coli*. Also, the *msc* gene was cloned into different expression vectors and expressed in different *E. coli* expression hosts under different osmotic conditions.

Recombinant TPSP, GT and the isolated TPP and TPS domain were purified to homogeneity. Under denaturing conditions the purified proteins exhibited molecular masses of 81 kDa (TPSP), 45 kDa (GT), 50 kDa (TPS) and 33 kDa (TPP) (Figure S2 in File S1).

The *T. tenax* MSC could not sufficiently be expressed using different expression plasmids and *E. coli* hosts. In all cases growth of the expression host was strongly inhibited, which could partially be restored by increasing the osmolarity of the medium (i.e. 300 mM KCl, 300 mM NaCl) (Figure S3 in File S1). However, higher osmolarity of the medium did not result in detectable overexpression levels of the recombinant MSC. These results suggest an effect of *T. tenax* MSC on the osmotic homeostasis of the expression host *E. coli* as reported previously for an archaeal MSC from *Methanocaldococcus jannaschii* [36] and support the notion that the membrane protein might function as mechanosensitive channel.

### Enzymatic properties of recombinant TPSP and artificial TPS and TPP domain

**Figure 2:**
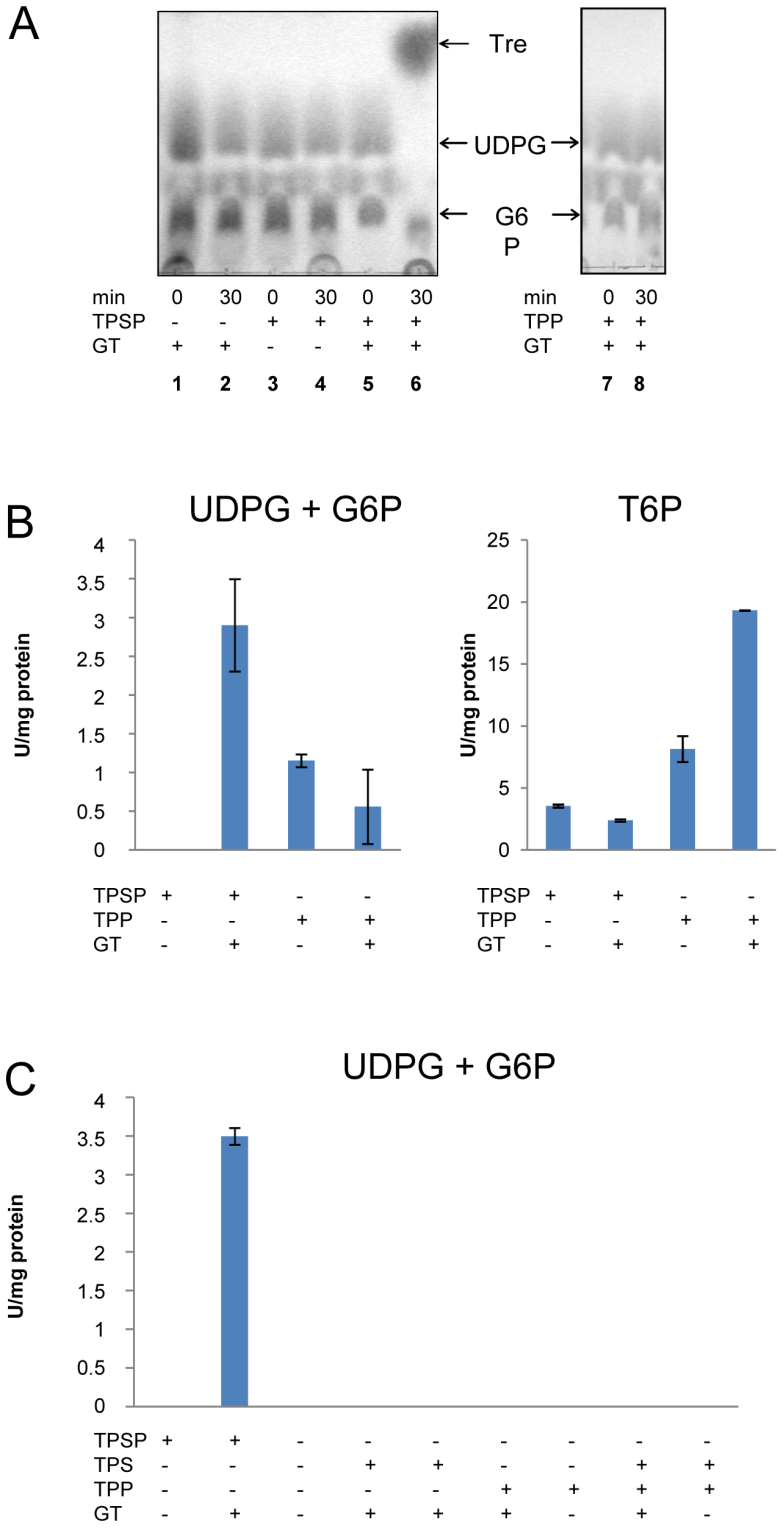
Trehalose and phosphate forming activity of the recombinant TPSP. (**A**) Trehalose formation from UDPG (uridine diphosphate-glucose) and G6P (glucose-6-phosphate) by TPSP and artificial TPP domain in combination with and without GT determined by thin layer chromatography on silica plates. (**B**) Specific activity of *T. tenax* TPSP and TPP at 80°C in the presence and absence of GT measured as P_i_ (inorganic phosphate) release from UDPG and G6P, i.e combined TPS and TPP activity (left panel) as well as from T6P (trehalose-6-phosphate), i.e. TPP activity, (right panel), respectively, via the malachite green assay. (**C**) Specific TPS activity of the *T. tenax* TPSP, TPS and TPP at 80°C in the presence and absence of GT measured as UDP release from UDPG and G6P in a discontinuous assay system. UDP was determined spectrophotometrically by monitoring the decrease in absorption due to oxidation of NADH via PK and LDH at 340 nm. (+) indicates the presence, (−) the absence of the respective protein.

TPSP activity was determined at 80°C (near the temperature optimum of *T. tenax* of 86°C [21]) in the presence of the substrates UDP-glucose (UDPG, 4 mM) and glucose-6-phosphate (G6P, 8 mM) as well as MgCl_2_. However, neither trehalose nor T6P formation was observed via TLC (lanes 3–4, Figure 2A) and also, no P_i_ formation could be detected using the malachite green assay (Figure 2B, left panel). UDP formation from UDPG and G6P could also not be observed, indicating that the fused TPSP protein does neither exhibit overall TPSP (trehalose or P_i_ formation from UDPG and G6P) nor TPS activity (T6P or UDP formation from UDPG and G6P) under assay conditions used. Also, the artificial TPS domain did not show any activity (Figure 2C).

However, the fused TPSP exhibited TPP activity (3.5 U/mg) as demonstrated by P_i_ formation from T6P (8 mM) and TPP activity could also be confirmed for the artificial TPP domain (8 U/mg) (Figure 2B, right panel). Taken together the results demonstrate TPP activity but no TPS activity for the *T. tenax* TPSP protein.

### Interaction of TPSP and GT *in vivo* - The prokaryotic (archaeal) trehalose synthase complex

The possible functional association of TPSP with GT in trehalose formation was investigated by the addition of recombinant, purified GT protein to the TPSP activity assay. In the presence of GT, trehalose formation from UDPG and G6P was detected (Figure 2A, lane 5–6). The specific P_i_-forming activity from UDPG and G6P was determined to be 2.9 U/mg. These results indicate that GT is essential for trehalose formation from UDPG and G6P by TPSP. Furthermore, also TPS activity of TPSP could only be detected in the presence of GT. The specific UDP forming activity from UDPG and G6P was 3.5 U/mg measured with partially purified TPSP and GT and therefore in the same range as the overall TPSP activity indicating that the TPS activity is the bottleneck in trehalose formation from UDPG and G6P via TPSP. However, the artificial TPS domain did not show any activity independent of the presence or absence of GT. Thus, the domain fusion of TPS and TPP is a prerequisite for GT mediated activation of TPS activity.

Both trehalose-6-phosphate synthase (TPS) and GT are members of glycosyltransferase families (family 20 and family 4, respectively (http://www.cazy.org/)). Consequently, it was necessary to demonstrate that GT does not substitute for the TPS activity. As shown in Figure 2C no TPS activity, i.e. UDP formation from UDPG and G6P, could be observed with GT alone. Also, no trehalose formation from G6P and UDPG was detected in the presence of GT and the artificial TPP domain using TLC (Figure 2A, lanes 7, 8) showing that GT does not simply substitute for TPS activity. However, P_i_ formation from UDPG and G6P could be observed with the TPP domain independently of the presence of GT (0.5–1 U/mg) (Figure 2B, left panel). It can be assumed that due to the missing trehalose formation this basal P_i_-forming activity is caused by unspecific phosphatase activity of the single TPP domain with G6P as substrate not observed for the fused TPSP. Thus, these studies confirm that GT does not simply substitute for the TPS activity and that the TPS domain of TPSP is required for trehalose forming activity.

The TPP activity of full-length TPSP was only slightly altered by GT whereas the TPS activity of TPSP strictly relies on the presence of GT. This indicates that GT primarily stimulates the TPS activity of TPSP. However, the TPP activity of the artificial TPP domain was significantly stimulated by GT (∼20 U/mg) (Figure 2B, right). Also the artificial TPP domain exhibited higher TPP activities than the TPSP fusion enzyme both in presence and absence of GT possibly indicating an inhibitory effect of the TPS on the TPP domain in the fusion enzyme.

**Figure 3:**
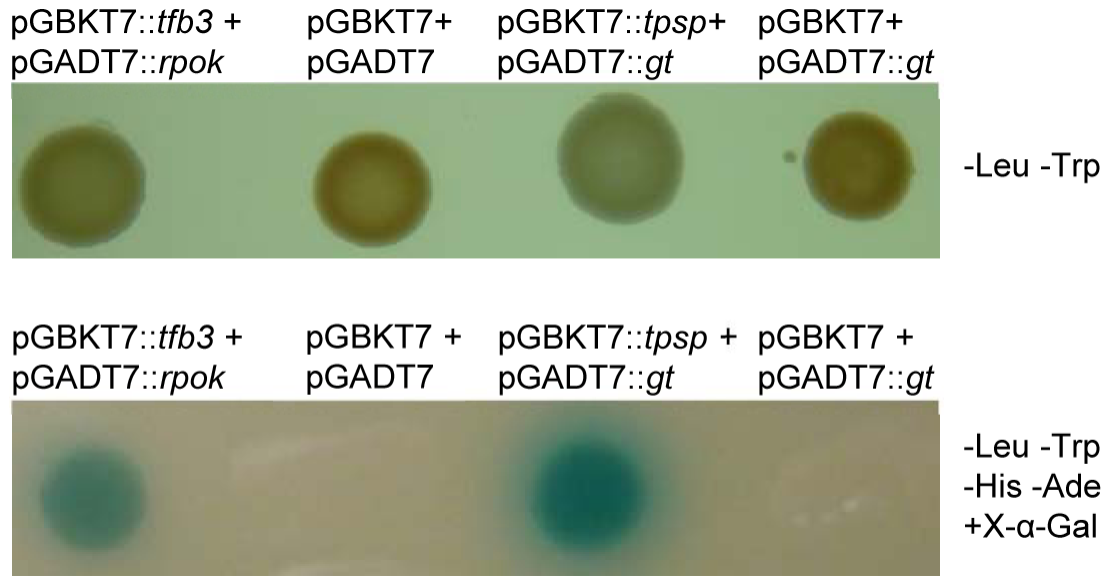
Complex formation of TPSP with GT *in vivo*. Yeast two-hybrid analysis of GT and TPSP interactions. Yeast strains (AH109) were co-transformed with pGADT7::*tpsp* and pGBKT7::*gt* and grown in SD medium (+2% (w/v) glucose, -leucine, -tryptophan) to an OD_600_ of 1.5 and diluted to an OD_600_ of 0.1. 5 µl of this suspension were dropped on SD-agar plates. **Lower panel**: -leucine, -tryptophan, -histidine, -adenine, + X-α-Gal. **Upper panel**: -leucine, -tryptophan. The plates were incubated for three days at 30°C. As positive control pGADT7::*rpok* and pGBKT7::*tfb3* were used [26]. The negative controls are the empty vectors pGBKT7 and pGADT7 and the false positive controls are the empty vectors pGBKT7 and pGADT7::*gt*.

To further test the potential for TPSP-GT interaction *in vivo*, yeast two-hybrid experiments were performed. Yeast cells transformed with the pGADT7::*TTX_1305* (GT, bait) and pGBKT7::*TTX_1304* (TPSP, prey) constructs formed blue colonies on X-α-gal agar plates (Figure 3), confirming that *T. tenax* TPSP and GT interact *in vivo* in *S. cerevisiae* and thus, most likely also form a complex in their native state in *T. tenax*.

This is further supported by the finding that high trehalose formation from UDPG and G6P and thus, bifunctional TPSP activity was observed in cell-free extracts of *T. tenax* via TLC (Figure S4 in File S1), indicating that the TPS/P pathway is operative and that the TPSP-GT complex is formed *in vivo* in *T. tenax*.

## Discussion

### Gene structure and genomic organization of TPSP proteins

**Figure 4:**
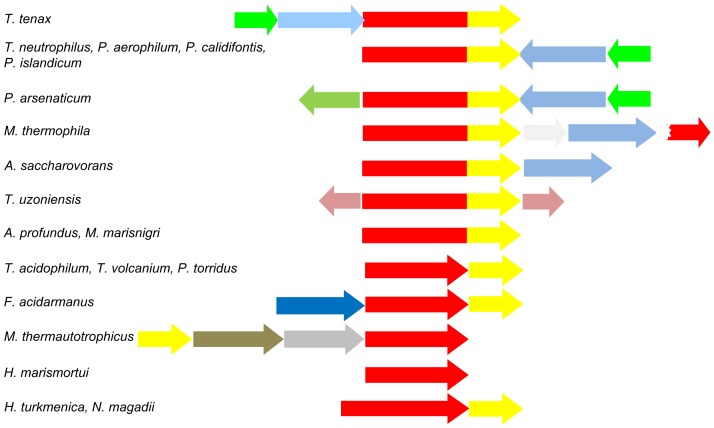
Comparative genomic context analyses of the genes encoding the TPS/P pathway via TPSP-fusions as well as single domain TPS and TPP proteins within the Archaea. Gene distributions and genomic organizations were analyzed using blast and genome region comparison tools at NCBI (http://blast.ncbi.nlm.nih.gov/) and IMG 2.0 (http://img.jgi.doe.gov/cgi-bin/w/main.cgi). As template the TPSP of *T. tenax* was chosen. Each arrow represents one gene with the arrow direction indicating the 5′-3′ orientation. Genes are drawn to scale. The genome region illustration is based on the 5′-3′ orientation of the *tps(p)* gene in the respective organism. Gene colors: *tps* (red); *tpp* (yellow), *gt* (light blue); *msc* (green), unknown (white), *glucose dehydrogenase* (pink), *glycoside hydrolase* (dark blue), *phosphomannomutase* (brown), *mannose-1-phosphate guanyltransferase* (grey). The broken red arrow represents an N-terminal *tps* gene fragment in *M. thermophila*.

TPSP fusion proteins are unusual in Archaea, since their occurrence in so far sequenced archaeal genomes is restricted to the Thermoproteales (except *Caldivirga maquilingensis* and *Thermofilum pendens* which lack the TPS/P pathway) and to *Methanosaeta thermophila*, *Methanocellus marisnigri*, *Acidilobus saccharovorans* as well as *Archaeoglobus profundus* (Figure 4). All other Archaea harboring the TPS/P pathway for trehalose biosynthesis contain separated TPS and TPP enzymes, e.g. *Thermoplasma acidophilum* for which the crystal structure of the TPP enzyme has been solved [10]. Also, in Bacteria the separated TPS and TPP enzymes are widespread. However, a variety of TPSP fusion proteins were identified recently, mostly from the Bacteroidetes group, some Cyanobacteria and Proteobacteria. One of these bacterial TPSP fusion enzymes was functionally characterized from *Cytophaga hutchinsonnii* by complementation studies in yeast [16]. From Eukaryotes the fused TPSP structure is well known, e.g. from the trehalose synthesizing complex of *S. cerevisiae* as well as from the plants *Arabidopsis thaliana* and *Selaginella lepidophylla* [11], [12], [37]–[40].

The operon organization of the *tpsp*, *gt* and *msc* genes in *T. tenax* is unique to Thermoproteales of the genera *Thermoproteus* and *Pyrobaculum* (Figure 1, Figure 4). All other archaeal and bacterial genes encoding TPSP fusions or single TPS and TPP do not show a comparable operon structure.

### Bifunctionality of TPSP

The intrinsic bifunctionality (TPS and TPP activity) of the *T. tenax* TPSP in the presence of the activator protein GT is in accordance with its protein sequence: The TPS domain exhibits high conservation in those residues shown to take part in catalysis and substrate binding in the *E. coli* OtsA protein (Figure S5 in File S1) [41], [42]. Also, the three described motifs in TPP enzymes harboring residues involved in active site formation are well conserved in the bifunctional *T. tenax* enzyme (motif 1: DXDX(T/V), motif 2: SG and motif 3: K(X)16–30(G/S)(D/S)XXX(D/N); all indicated in Figure S5 in File S1) [10], [11]. In contrast, in those eukaryotic TPSPs described from yeast and plants (e.g. *S. cerevisiae* and *S. lepidphylla*) exhibiting only TPS activity the TPP motifs are missing or only partially present and vice versa. The enzymatically inactive TPSPs fulfilling regulatory/structural implications both the TPS residues and TPP motifs are incomplete or absent (Figure S5 in File S1) [11].

The TPS domain of TPSP showed an inhibitory effect on its TPP activity which cannot be explained at the sequence level. It seems likely that this inhibition might be due to structural effects of the domain fusion causing e.g. a decreased flexibility or substrate accessibility in the TPP domain.

Bifunctional activity has so far only been described for the TPSP fusion from *C. hutchinsonii* which has been shown to complement for both, Tps1 and Tps2 in yeast mutants. This enzyme also shows high conservation in the substrate binding residues and motifs [16]. However, in contrast to the *T. tenax* TPSP the *C. hutchinsonii* fusion enzyme has not been described to rely on an additional, activating protein like GT.

### TPSP-GT complex formation in *T. tenax*

*In vitro* and *in vivo* studies revealed that trehalose formation from G6P and UDPG by *T. tenax* TPSP strictly relies on the presence of GT and that TPSP activation by GT is mediated by complex formation rather than protein modification. Furthermore, enzymatic studies demonstrate that GT solely activates the TPS activity of TPSP which strictly depends on the presence of GT, whereas the TPP activity of the fusion enzyme was only slightly affected by GT. Furthermore, the artificial single domain TPS did not show any activity independent of the presence or absence of GT and the artificial single TPP domain. Together with the protein interaction of TPSP and GT shown by yeast two-hybrid analyses these results suggest that the GT mediated activation of TPS activity via complex formation also relies on the domain fusion of TPS and TPP.

To our knowledge this is the first report of a trehalose synthase complex in Prokaryotes. In Eukaryotes, complex formation of trehalose forming enzymes is so far only known from *S. cerevisiae*. The yeast complex is composed of four proteins: *Sc*Tps1 (TPS activity and non-fused TPS structure), *Sc*Tps2 (TPP activity, fused TPSP structure) and two subunits (*Sc*Tsl1, *Sc*Tps3, both fused TPSP structure) with regulatory and structural implications [17]. Thus, compared to the yeast complex the results reported herein indicate that the *T. tenax* TPSP-GT complex is much more simple in structure/composition and therefore maybe primordial.

*Sc*Tps1 was described to exist both in its complex bound and also in its free form in yeast [17] and the TPS activity of the free form is significantly reduced (∼20%). Both forms fulfill different functions and are differentially regulated by phosphate, which triggers T6P and trehalose levels in the cell. This in turn influences growth on glucose and fructose as well as glucose sensing and influx into glycolysis [17], [18], [43]–[45]. Therefore, *Sc*Tps1 activity is profoundly regulated by complex formation/dissociation that provides an intriguing regulatory capability allowing for fine tuning of metabolic processes. It is tempting to speculate that a similar mechanism of complex formation and dissociation allows the regulation of TPS activity of TPSP as well as of metabolic processes in *T. tenax* and members of the Thermoproteales and even in other prokaryotes harboring fused TPSP proteins. Similarly to the *T. tenax* TPSP, TPP activity of the *C. hutchinsonii* TPSP fusion has been reported to be approximately 1,000-fold higher than its TPS activity [16]. Thus, the significantly decreased activity of the TPS compared to TPP might be a general feature of prokaryotic TPSP fusions and reversible complex formation by so far unknown GTs could be a more general strategy to trigger TPS activity of fused TPSPs and thus trehalose synthesis in Prokaryotes.

### Phylogenetic aspects of TPSP

**Figure 5:**
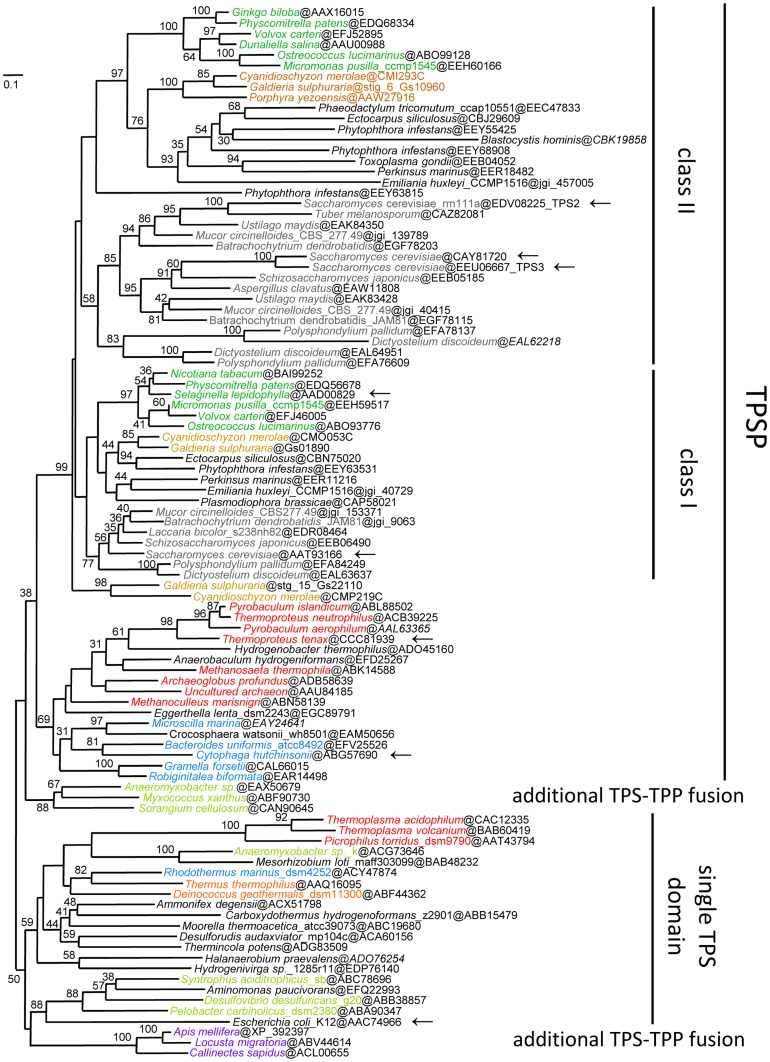
Maximum likelihood phylogenetic analysis of the TPS domain of fused TPSP and single domain TPS proteins. The phylogenetic tree was inferred by RAxML with a LG+F+Γ4 model of sequence evolution based on 99 sequences and 203 positions. All TPSP sequences located in the upper part of the tree correspond to enzymes with fused TPS+TPP domains. The non-fused single TPS domain sequences are clustering in the lower part of the tree. Bootstrap (BS) values are only indicated at internal branches if they are 30% or higher. The following groups were colored: unikonts (grey); red algae (brown), green plants (green); Archaea (red); Bacteroidetes (blue); Delta-Proteobacteria (lemon); Deinococci-Thermus (orange) and Pan-Crustacean (magenta). The scale bar indicates the mean number of inferred substitutions per site.

Phylogenetic analyses of the TPS region of the fused TPSP proteins and single TPS enzymes revealed two main monophyletic lineages of fused TPSP proteins, clearly separated from the bacterial single domain TPS enzymes as illustrated in the maximum likelihood (ML) and in the Bayesian phylogenetic tree (Figure 5, Figure S6 in File S1):

(i) The vast majority of the fused eukaryotic sequences (56 out of 59) form a monophyletic group (bootstrap support (BS) 99%), with two sub-trees corresponding to the class I (similar to yeast *Sc*Tps1) and class II (similar to yeast *Sc*Tps2) form of the fused TPSP enzymes, which are therefore the result of an early gene duplication in the common ancestor of extant Eukaryotes. The absence of animal sequences in the group of eukaryotic sequences, suggests two independent and rather late losses of class I and class II proteins after the separation of fungi. (ii) Also, the fused prokaryotic TPSP sequences (17 out of 20 in Figure 5) form a monophyletic group (BS 69%). A subsequent phylogenetic analysis (see Figure S7a and b in File S1) focusing on the fused prokaryotic sequences shows that the majority of these fused sequences (38 out of 70) are from the bacterial phylum Bacteroidetes which represents the only consistent larger taxonomic group within the fused prokaryotic TPSP proteins. The twelve archaeal sequences including the *T. te*nax TPSP are usually closely related to bacterial ones and there is a complete lack of meaningful archaeal relationships beyond the family level (i.e. Thermoproteaceae), thus suggesting the rather recent origin (in terms of global archaeal evolution) of these enzymes via HGT from bacteria. In addition, the non-Bacteroidetes bacterial sequences do not display any meaningful higher order phylogenetic relationships. This suggests that at least the fused prokaryotic sequences including the archaeal TPSPs likely originated in a gene fusion event in the phylum Bacteroidetes with many independent subsequent horizontal gene transfer events (HGTs) to other bacterial and archaeal lineages. This scenario is supported by the following observations, (i) there are only two non-fused Bacteroidetes sequences, i.e. *Rhodothermus* and *Salinibacter*, which represent the sistergroup to the four canonical Bacteroidetes classes and (ii) these sequences were very likely acquired by the common ancestor of these two species in an HGT event with an ancient representative of the order Thermales (phylum Deinococcus/Thermus) (for a more complete picture of the evolution of single TPS enzymes see also Figure S8a and b in File S1). In contrast to this evolutionary scenario, a recent study explained the patchy distribution of bacterial and archaeal sequences via several independent fusion events, rather than as the result of independent HGTs [16]. However, the obtained tree topologies and conclusions including the proposition of a bacterial origin of the fused class I and II eukaryotic TPSP enzymes were comparable to those presented here.

Based on our analyses a deltaproteobacterial origin of the fused prokaryotic sequences cannot be strictly excluded, since the fused deltaproteobacterial sequences, which cluster together (BS 88%), assume an intermediary position between the fused TPSP and the single domain TPS enzymes within both the ML and the Bayesian tree (Figure 5, Figure S6 in File S1). However, there are several reasons that argue against this hypothesis: (i) The fused sequences are limited to the Myxococcales, a single out of more than ten orders within the class Deltaproteobacteria. (ii) The relationship of fused TPSPs within this group significantly differs from their 16S rRNA based taxonomic status, i.e. the *Anaeromyxobacter* (Cystobacterinae) TPSPs are more closely related to the *Haloangium* sequence (Nannocystinae) than to the other Cystobacterinae sequences from *Myxococcus* and *Stigmatella* (BS 74%, posterior probability (PP) 0.99). Conversely, a phylogenetic study on the β subunit of the RNA polymerase confirms the 16S rRNA based phylogeny (data not shown). (iii) The less long-branch attraction (LBA) sensitive Bayesian analysis indicates a closer relationship of these sequences to the single domain TPSs, which also include several non-fused deltaproteobacterial sequences. Hence, an origin of these fused deltaproteobacterial TPSP in either an additional, independent fusion event or an ambiguous position due to striking acceleration of their evolutionary rates after HGT events seems more likely than that these sequences represent the ancestor of either the two major fused eukaryotic and prokaryotic TPSP or of only the prokaryotic TPSP.

An additional minor group of fused TPSP proteins, which likely originated in an additional independent fusion event is represented by pan-crustacean (insects and crustaceans) sequences (Figure 5, Figure S6 in File S1), which cluster together with the prokaryotic single domain TPS enzymes in both the ML and PhyloBayes analyses (BS 50%, PP 0.84). However, both, the fused deltaproteobacterial and the pan-crustacean groups, neither belong to the two major monophyletic TPSP clusters, nor are they specifically related to each other, thus rendering their independent origin in additional fusion events even more likely.

The overall topology of the ML tree is compatible with a monophyletic origin of the fused eu- and prokaryotic TPSPs, however the support is very low (BS 38%, PP 0.68). Given the distribution of fused prokaryotic TPSP sequences the fusion event of TPS and TPP to TPSP must have happened early in evolution, at least before the last common ancestor of extant eukaryotes. In light of the above, the most likely scenario seems a bacterial origin with a fusion that occurred early during the evolution of the phylum Bacteroidetes, however the great age of this event together with an abundance of HGT events, which are usually associated with a clear acceleration of the evolutionary rate of the corresponding sequences (i.e. long branches) is interfering with a highly supported phylogenetic inference of this scenario. However, a very similar tree topology (monophyly of fused pro- and eukaryotic sequences) as shown in Figure 5 for the TPS domains had been published for the TPP domains of single and fused TPP and TPSP sequences inferred by MrBayes [16]. These analyses obtained also a high support and were confirmed by NJ and ML analyses.

### Role of trehalose in *T. tenax* - Model of stress response

Despite the presence of trehalose shown for many Archaea [3], [46] and the existence of multiple trehalose synthesis pathways (see Figure S1 in File S1), the role of trehalose in Archaea still remains unknown.

However, some hints point to an exclusive function of trehalose in stress adaptation rather than as carbon source and/or carbon storage compound in *T. tenax* [24]: (i) *T. tenax* is unable to grow on trehalose and measurements in crude extracts revealed that this organism is unable to degrade the disaccharide. (ii) No homologs of known trehalose degrading enzymes, e.g. bacterial trehalases, could be identified in the genome. (iii) Only the unidirectional TreT pathway for trehalose synthesis is present in addition to the TPS/P pathway. (iv) Glycogen was shown to represent the carbon storage compound rather than trehalose [47]. (v) TPSP in *T. tenax* is organized in an operon together with a putative mechano-sensitive channel (MSC) as shown in this study.

MSC have been discovered in organisms belonging to all three domains of life [36], [48]. They open in response to membrane tension releasing hydrated solutes from the cell to allow for survival. The best studied function of bacterial MSC is the essential protective role in the regulation of the cell volume under osmotic stress conditions [49]. The role of MSC in Archaea and also in *T. tenax* has not yet been established, however they are supposed to fulfill similar functions [36].

**Figure 6:**
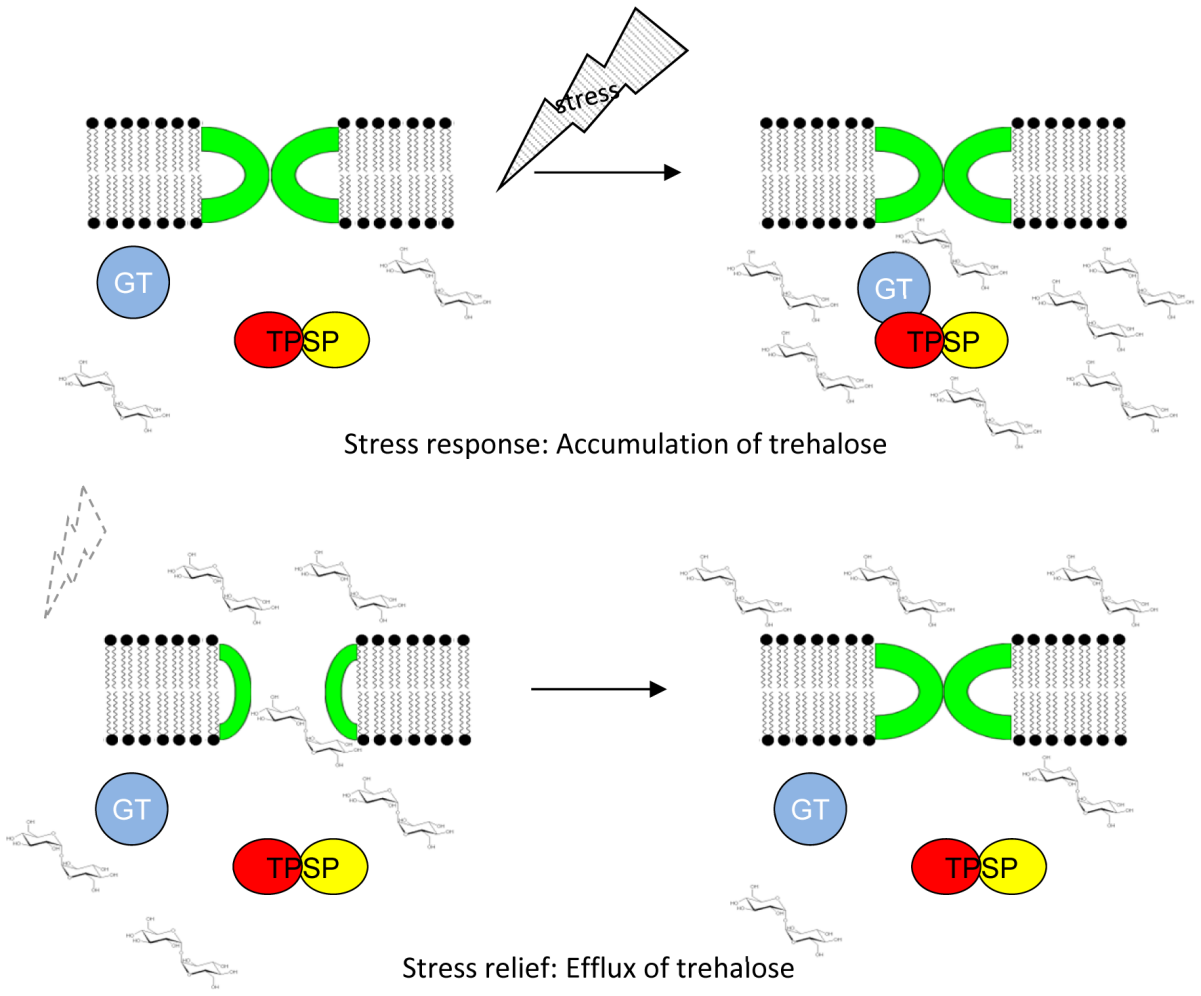
Proposed model of stress response in *T.* *tenax* involving TPSP (yellow, red), GT (blue) and MSC (green). In response to stress (e.g. high osmolarity), trehalose is synthesized via complex formation of TPSP and GT, resulting in an increased intracellular trehalose concentration (top right). Upon stress relief (e.g. hypo-osmotic shock) the MSC opens in response to changes in membrane tension resulting in trehalose efflux (down left).

Thus, on the basis of the presented results the following model of stress response involving the *msc-gt-tpsp* operon can be proposed for *T. tenax* (Figure 6): In response to stress caused e.g. by high osmolarity or temperature change, trehalose is synthesized via complex formation of TPSP and GT, resulting in an increased intracellular trehalose concentration. Upon stress relief (e.g. hypo-osmotic shock) the MSC opens in response to changes in membrane tension resulting in trehalose efflux. The ability to rapidly jettison trehalose instead of its enzymatic degradation prevents the cell from further swelling and bursting. It seems likely that this stress response represents a GT-mediated process. In future studies this model will be tested by detailed functional analyses of the activation of TPSP by GT via complex formation and of the MSC, in order to further unravel adaptation strategies of extremophilic organisms to changing environmental conditions.

## Supporting Information

File S1 **Supporting information.**
(PDF)
