# Supplementary material for: The First Prokaryotic Trehalose Synthase Complex Identified in the Hyperthermophilic Crenarchaeon Thermoproteus tenax
Source: PLoS One. 2013 Apr 23;8(4):e61354. doi: 10.1371/journal.pone.0061354 (PMC3634074; doi:10.1371/journal.pone.0061354)
Supplement: File S1 — Supporting information. (PDF) [file pone.0061354.s001.pdf]

SUPPLEMENTARY INFORMATION

Figure S1:

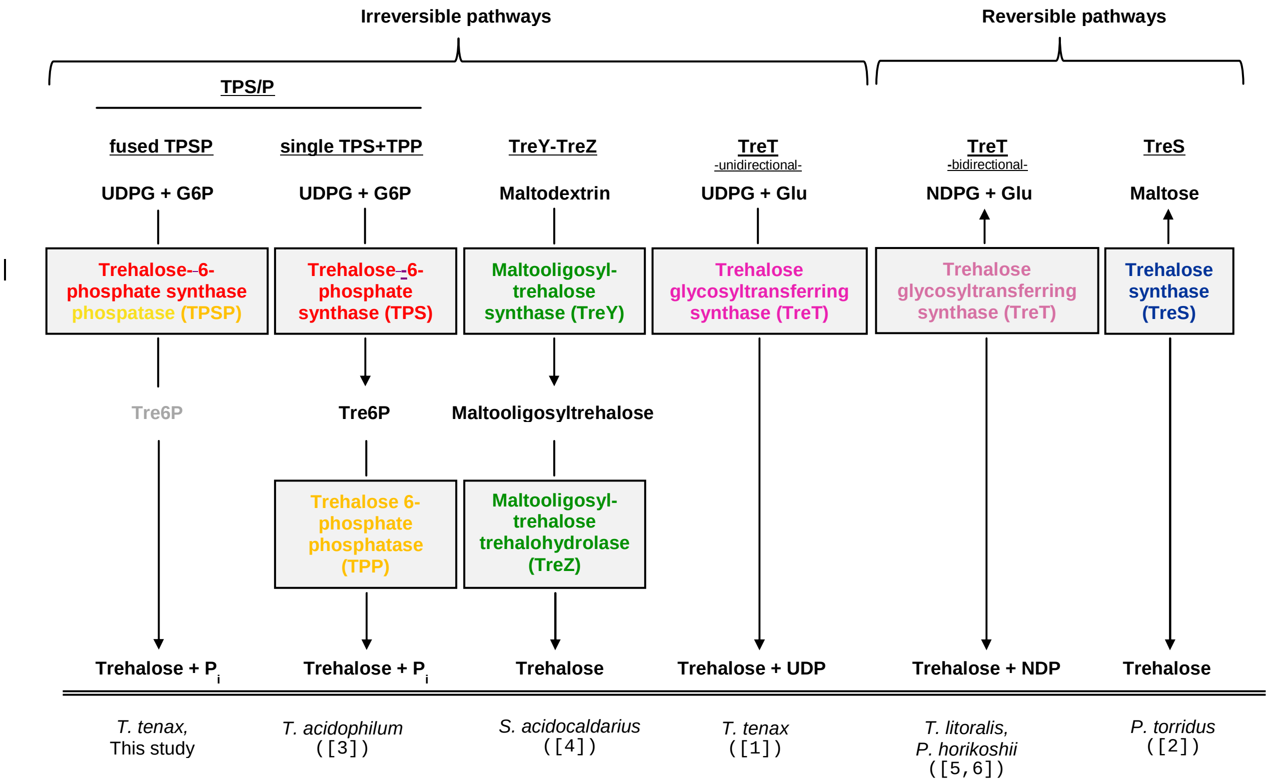

Figure S1: Schematic illustration of the irreversible (left) and reversible (right) trehalose synthesis pathways in Archaea.
Pathway names are underlined. The educts, intermediates and products of each pathways and the enzymes involved (highlighted in boxes) are shown. Examples of archaeal organisms utilizing the respective pathways are given. Abbreviations: UDPG (uridine diphosphate-glucose), G6P (glucose-6-phosphate), Glu (glucose), NDPG (nucleoside diphosphate glucose), T6P (trehalose-6-phosphate), P_i (inorganic phosphate).

Figure S2

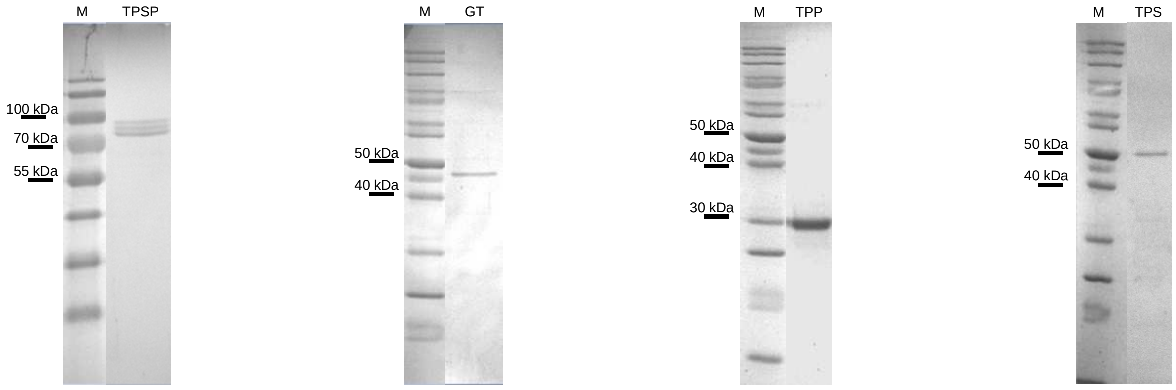

Figure S2: Purification of the recombinant trehalose-6-phosphate synthase/phophatase (TPSP), the recombinant putative glycosyltransferase (GT) and of the artificial trehalose-6-phosphate phosphatase (TPP) and the artificial trehalose-6-phosphate synthase (TPS) domain of TPSP.

Coomassie stained 12.5 % SDS gel electropherogram of the recombinant proteins after heterologous expression in *E. coli* and purification by heat precipitation, ion exchange chromatography (TPSP, TPP) or immobilized metal ion affinity chromatography (TPS, GT), respectively, and gelfiltation. M: protein standard.

Figure S3

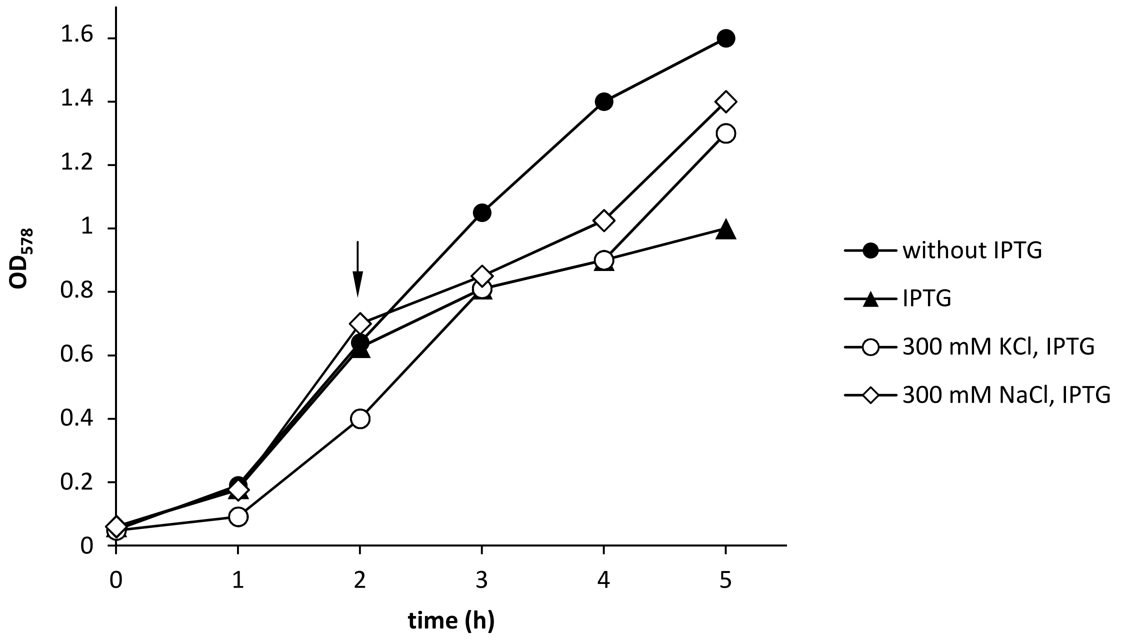

Figure S3: Effect of expression of the putative MSC of *T. tenax* on growing *E. coli* BL21 (DE3) pLys cultures.

Growth of *E. coli* BL21(DE3) pLys (▲) was inhibited after induction of *T. tenax* MSC expression by adding IPTG (1 mM) at OD_{578} ~0.6 (marked with an arrow). Growth was partially rescued in media of high osmolarity containing either 300 mM KCl (○), 300 mM NaCl (◊). Growth of non-induced BL21(DE3) pLys culture, harbouring pET24a-*msc*C-his (●) served as control.

Figure S4

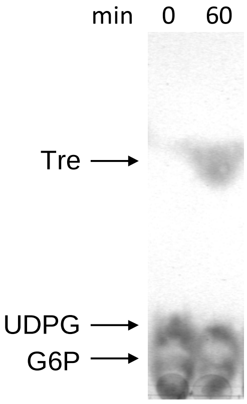

Figure S4 Trehalose formation from UDPG (uridine diphosphate-glucose) and G6P (glucose-6-phosphate) at 86°C by cell-free extracts of *T. tenax* (50 µg of protein).
Reaction mixture incubation, substrate and product determination by thin layer chromatography on silica plates were carried out as described in Material and Methods.

Figure S5

```
T_ten_TPSP    1 --------------MRLIVVSNRLPVTIS--PSG--EI-RESVGGLATAMKS------FLGAVNGGREL
C_hut_TPSP    1 -----------MNDEKRLIIVAYRIPFKVT-FKESGERVLQQNSGGLVSAILS------LAEKGDVFGKN
S_cer_TPS1    1 MTTDNAKAQLTSSSGGNIIVVSNRLPVTITKNSSTGQYEYAMSSGGLVTALEG------LKKTYTFKWFG
S_cer_TSL1  302 VSDLEMDDAKQDYKVPKFGGYSNKSKLKKYALLRSSQELFSRLPWSIVPSIKGNGAMKNAINTAVLENII
S_cer_TPS3  273 -SDLET-DATKKYNVPKFGGYSNNAKLRAS-LMRNSYELFKHLPWTIVDSDKGNGSLKNAVNIAVAEKTV
S_cer_TPS2   29 LGESNDDWKISATTGNSALYSSLEYLQFDSTEYEQHVVGWTGEITRTERNLFTREAKEKPQDLDDDPLYL
S_lep_TPSP   89 PSLSDEPSKISSGRGQRLLVVANRLPLSATRKGETEWNL-EMSAGGLVSALLG-------VKQFEVTWIG
E_coli_TPS    1 -------------MSRLVVVSNRIAPPDE--HAA-------SAGGLAVGILG------ALKAAGG----
T_acid_TPS    1 --------------MKYIVVSSRCPFSHD--KAAGKEIIKENVGGVATALRR------AMEKYGG----

T_ten_TPSP   45 GLEEVVWVGWSGVPSERESNDLRERL-----RGMGLEPVPLSSEEVEGFYEGFSNSTLWPLFHGF----S
C_hut_TPSP   53 --QKIHWFGYS-TPDILQEGETQVEN-----ENFIVHPVLIEDALNDAYYEGFCNSTIWPISHYY----P
S_cer_TPS1   65 ------WPGLE-IPDDEKDQVRKDLL-----EKFNAVPIFLSDEIADLHYNGFSNSILWPLFHYH----P
S_cer_TSL1  372 PHRHVKWVGTVGIPTDEIPENILANISDSLKDKYDSYPVLTDDVTFKAAYKNYCKQILWPTLHYQIPDNP
S_cer_TPS3  340 KEP-VSWVGTMGIPTDELPHEVCHKISKKLEQDFSSFPVVTDDITFKGAYKNYAKQILWPTLHYQIPDNP
S_cer_TPS2   99 TKEQINGLTTTLQDHMKSDKEAKTDTTQTAPVTNNVHPVWLLRK-NQSRWRNYAEKVIWPTFHYILN--P
S_lep_TPSP  151 ------WPGVY-VQDEKGEKSLRGAL-----EEKGFVPVLLDEATVDQYYNGYCNNVLWPLFHYIGL--R
E_coli_TPS   38 -----LWFGWSGETGNEDQPLKKVKK-----GNITWASFNLSEQDLDEYYNQFSNAVLWPAFHYR----L
T_acid_TPS   44 -----TWICWG--DGKLDGKYLDEDV-----GKYRIRRVMLSVPEKHGYYDLYSNRTLWPLFHYF----R

T_ten_TPSP  106 EYATYEEK-----HWRAYRGVNEKYAKAVVALARPGDLVWIHDYHLMLAPAIVR------EAAEVGVGFF
C_hut_TPSP  111 YLTSFNEE-----DYEAYVKANKIFADQINAFIRPGDMVWIQDYQLMLLPGMLR-----SENPDASIGYF
S_cer_TPS1  119 GEINFDEN-----AWLAYNEANQTFTNEIAKTMNHNDLIWVHDYHLMLVPEMLRVKIHEKQLQNVKVGWF
S_cer_TSL1  442 NSKAFEDH-----SWKFYRNLNQRFADAIVKIYKKGDTIWIHDYHLMLVPQMVR-----DVLPFAKIGFT
S_cer_TPS3  409 NSKAFEDH-----SWDYYQKVNQKFSDRIVSVYKPGDTIWIHDYHLMLVPQMVR-----EKLPKAKIGFF
S_cer_TPS2  166 SNEGEQEK----NWWYDYVKFNEAYAQKIGEVYRKGDIIWIHDYYLLLLPQLLR---MKFNDESIIIGYF
S_lep_TPSP  207 QEDRLAATRSLLSQFNAYKRANRLFAEAVFNFYQEGDVVWCHDYHLMFLPSYLK-----EKDSQMKVGWF
E_coli_TPS   94 DLVQFQRP-----AWDGYLRVNALLADKLLPLLQDDDIIWIHDYHLLPFAHELR-----KRGVNNRIGFF
T_acid_TPS   98 ERVKYTDN-----GYEIYRRVNEKFTNMIVQSLSPEDVIWIHDYQLSLVPKMLR-----DRGITNRIIFT

T_ten_TPSP  165 LHIPFPPAELLQLLPSEWRREILEGLLGSDLVGFHTYEYSANFSRSVVRFLGYKVE-MGAIAVGH-----
C_hut_TPSP  171 HHIPFATFEIFRLLPKQWQTELLKGMIGADLVGFHTNDYSQYFLRSVQRVLGYDTT-ARDISIPN-----
S_cer_TPS1  184 LHTPFPSSEIYRILP--VRQEILKGVLSCDLVGFHTYDYARHFLSSVQRVLNVNTL-PNGVEYQG-----
S_cer_TSL1  502 LHVSFPSSEVFRCLA--QREKILEGLTGADFVGFQTREYARHFLQTSNRLLMADVVHDEELKYNG-----
S_cer_TPS3  469 LHVSFPSSEVFRCLA--NRERILEGIIGANFVGFQTKEYKRHFLQTCNRLLAADVS-NDEVKYHC-----
S_cer_TPS2  229 HHAPWPSNEYFRCLP--RRKQILDGLVGANRICFQNESFSRHFVSSCKRLLDATAK-KSKNSSNSDQYQV
S_lep_TPSP  272 LHTPFPSSEIYRTLP--LRAELLQGVLAADLVGFHTYDYARHFVSACTRILGLEGT-PEGVEDQG-----
E_coli_TPS  154 LHIPFPTPEIFNALP--TYDTLLEQLCDYDLLGFQTENDRLAFLDCLSNLTRVTTRSAKSHTAWG-----
T_acid_TPS  158 WHIPWVSKEMFETLP--ESRDIIDSLSRSDFITFHTETYRKNFT---------DLL-DEN----------

T_ten_TPSP  229 ----RRVRVGVFPIGIDFDRFYNSSQDPSVVEEMAKLREMLGRAKVVFSIDRLDYTKGVLRRVAAWERFL
C_hut_TPSP  235 ----RIVRVDTFPISIDYNKFQELYNDPGVIEKRKEFLKDLSAEKNIFSVDRLDYSKGLIHRLNGFEYFL
S_cer_TPS1  246 ----RFVNVGAFPIGIDVDKFTDGLKKESVQKRIQQLKETFKGCKIIVGVDRLDYIKGVPQKLHAMEVFL
S_cer_TSL1  565 ----RVVSVRFTPVGIDAFDLQSQLKDGSVMQWRQLIRERWQGKKLIVCRDQFDRIRGIHKKLLAYEKFL
S_cer_TPS3  531 ----NIVSVMYAPIGIDYYHLTSQLRNGSVLEWRQLIKERWRNKKLIVCRDQFDRIRGLQKKMLAYERFL
S_cer_TPS2  296 SVYGGDVLVDSLPIGVNTTQILKDAFTKDIDSKVLSIKQAYQNKKIIIGRDRLDSVRGVVQKLRAFETFL
S_lep_TPSP  334 ----KNTRVAAFPVGIDSERFIEAVETDAVKKHMQELSQRFAGRKVMLGVDRLDMIKGIPQKLLAFEKFL
E_coli_TPS  217 ----KAFRTEVYPIGIEPKEIAKQAAGP-LPPKLAQLKAELKNVQNIFSVERLDYSKGLPERFLAYEALL
T_acid_TPS  206 ----SRAKSLVIPLGIDYRYFSKTRG------TDIKSYALMDR-KLIFSIDRLDYTKGLVNRVLSIEELL

T_ten_TPSP  295 REHPEWRGRAVFVLVVVPSR-TGVPMYEEMKRQIDREVGRINGELGELNW-VPIVYLYRFIPSPTLMALY
C_hut_TPSP  301 DQYPQWHTRVVLTMIVVPSR-ETILKYAEMKHELEEAVGRINGKYGTLGW-RPVIYLYRSLSFEELGALY
S_cer_TPS1  312 NEHPEWRGKVVLVQVAVPSR-GDVEEYQYLRSVVNELVGRINGQFGTVEF-VPIHFMHKSIPFEELISLY
S_cer_TSL1  631 VENPEYVEKSTLIQICIGSS-KDV-ELE---RQIMIVVDRINSLSTNISISQPVVFLHQDLDFSQYLALS
S_cer_TPS3  597 IENPEYIEKVVLIQICIGKS-SDP-EYE---RQIMVVVDRINSLSSNISISQPVVFLHQDLDFAQYLALN
S_cer_TPS2  366 AMYPEWRDQVVLIQVSSPTANRNSPQTIRLEQQVNELVNSINSEYGNLNF-SPVQHYYMRIPKDVYLSLL
S_lep_TPSP  400 EENSEWRDKVVLVQIAVPTR-TDVLEYQKLTSQVHEIVGRINGRFGSLTA-VPIHHLDRSMKFPELCALY
E_coli_TPS  282 EKYPQHHGKIRYTQIAPTSR-GDVQAYQDIRHQLENEAGRINGKYGQLGW-TPLYYLNQHFDRKLLMKIF
T_acid_TPS  265 RRHPDLVGKFVYVMIVTPSR-TTVSDYVAMKRELEMHIGRINGEFGSISW-MPILYMYRKISDKMLVSYY

T_ten_TPSP  363 NIADVALITPLRDGMNLVAKEFVASKRDC------RGVLILSELAGASKELA-EALVINPNDVGGTAEAI
C_hut_TPSP  369 TLADVALITPIRDGMNLVAKEFVATRTDK------KGVLILSEMAGASLELN-GAILINPTDKRTLAAAI
S_cer_TPS1  380 AVSDVCLVSSTRDGMNLVSYEYIACQEEK------KGSLILSEFTGAAQSLN-GAIIVNPWNTDDLSDAI
S_cer_TSL1  696 SEADLFVVSSLREGMNLTCHEFIVCSEDK------NAPLLLSEFTGSASLLNDGAIIINPWDTKNFSQAI
S_cer_TPS3  662 CEADVFLVDALREGMNLTCHEFIVSSFEK------NAPLLLSEFTGSSSVLKEGAILINPWDINHVAQSI
S_cer_TPS2  435 RVADLCLITSVRDGMNTTALEYVTVKSHMSNFLCYGNPLILSEFSGSSNVLK-DAIVVNPWDSVAVAKSI
S_lep_TPSP  468 AITDVLLVTSLRDGMNLVSYEFVACQKDK------KGALILSEFAGAAQSLGAGSILINPWNIIESSNAI
E_coli_TPS  350 RYSDVGLVTPLRDGMNLVAKEYVAAQDPAN-----PGVLVLSQFAGAANELT-SALIVNPYDRDEVAAAL
```

```
T_acid_TPS   333 KNADIALITPLIDGLNLVSKEFVASSD--------KGILILSRFAGASNCLD-GAVVVNPNSLGEVAEAI

T_ten_TPSP   426 AEALSMSEDEQCRRIRAMQERLRMRDVVRWGTDFIYSLISAKSAREEVEKALRYMEELSVDKLKSDFAKA
C_hut_TPSP   432 IQALTMDEQEQTQRMQMMQDRIRVYDIFKWTEDYMDQLDIIKLKQMEFEEKIINQDVLKSFKQIFD--ES
S_cer_TPS1   443 NEALTLPDVKKEVNWEKLYKYISKYTSAFWGENFVHELYS-----------------TSSSSTSSSATKN
S_cer_TSL1   760 LKGLEMPFDKRRPQWKKLMKDIINNDSTNWIKTSLQDIHISWQFNQEGSKIIKLNTKTLMEDYQSS--KK
S_cer_TPS3   726 KRSLEMSPEEKRRRWKKLFKSVIEHDSDNWITKCFEYINNAWESNQETSTVFNLAPEKFCADYKAS--KK
S_cer_TPS2   504 NMALKLDKEEKSNLESKLWKEV--PTIQDWTNKFLSSLKEQASSDDDMERKMTPALNRPVLLENYK--QA
S_lep_TPSP   532 ADALNMPEEEREERHRHNFMHITTHSAQVWAETFISELNDSILEAELRTLHIPPQLPLDKAVAKYSESKN
E_coli_TPS   414 DRALTMSLAERISRHAEMLDVIVKNDINHWQECFISDLKQIVPRSAESQQRDKVATFPKLA*
T_acid_TPS   394 YSAMNMGEDEIRERLRKMKEEVSRRDTDWWIRR-INAIAKRKINDRTVT*
E_coli_TPP     1 ---------------------------------------------------------MTEPLTETPELS
T_acid_TPP     1 ------------------------------------------------------MIEQSLEDALYAIVT

                      Motif I                                   Motif II

T_ten_TPSP   496 KRRLLLLDYDG--------TLVPHYPYPHMAVPDGDLLELLSRLAALPETAVYVVSGRGRDFLDGWLGRL
C_hut_TPSP   500 SRRLFLLDYDG--------TLSPIVPDPSSSKPSVGIVDVLKKLSSDFNVNVAIISGRDKEFLN-YIFDP
S_cer_TSL1   828 RMFVFNIAE----------------------PPSSRMISILNDMTSKGNIVYIMNSFPKPILENLYSRVQ
S_cer_TPS3   804 HLFIFKISE----------------------PPTSRMLSLLSELSSN-NIVYVLSSFTKNTFESLYNGVL
S_cer_TPS2   570 KRRLFLFDYDG--------TLTPIVKDPAAAIPSARLYTILQKLCADPHNQIWIISGRDQKFLNKWLGGK
S_lep_TPSP   602 RLVILGFNSTLTAQVEAPRGRAPDQIREMKIRLHPSIKDILNVLCSDPKTTIVILSGSERVALDEVFGEF
E_coli_TPP    13 AKYAWFFDLDG--------TLAEIKPHPDQVVVPDNILQGLQLLATASDGALALISGRSMVELDALAKPY
T_acid_TPP    16 QNPMIFLDYDG--------TLVPIIMNPEESYADAGLLSLISDLKERFDT--YIVTGRSPEEISRFLP-L

T_ten_TPSP   558 P--VGLVAEHGFFLKHPG------GEWKSLG--KVDPSWRQYAKGIMEDFASNVPGSFVEVKEAGIAWHY
C_hut_TPSP   561 N--TILSAEHGAFLRIPG------QDWELQY--DYNDQWKRSVLPVFQKYTDRCAGSFIENKSTSLAWHY
S_cer_TSL1   876 N--IGLIAENGAYVSLNG-------VWYNIV---DQVDWRNDVAKILEDKVERLPGSYYKINESMIKFHT
S_cer_TPS3   841 N--IGLIAENGAYVRVNG-------SWYNIV---EELDWMKEVAKIFDEKVERLPGSYYKIADSMIRFHT
S_cer_TPS2   632 LPQLGLSAEHGCFMKDVS------CQDWVNLTEKVDMSWQVRVNEVMEEFTTRTPGSFIERKKVALTWHY
S_lep_TPSP   672 D--LWLAAENGMFLRHTQ------GEWMTTMPEHLNMDWLESVQLVFDYFCERTPRSFVETRETSLVWNY
E_coli_TPP    75 R--FPLAGVHGAERRDIN------GKTHIVH--LPDAIARDISVQ-LHTVIAQYPGAELEAKGMAFALHY
T_acid_TPP    75 D--INMICYHGACSKINGQIVYNNGSDRFLG--VFDRIYED-----TRSWVSDFPGLRIYRKNLAVLYHL

                                                                        Motif III

T_ten_TPSP   618 --RNADETIAEKAVVELIDALSNAL-AGSGLSILRGKKVVEVRPAGYTKGTAAKMLLDELSP--------
C_hut_TPSP   621 --RNTEKEYAHIRSREFIEELENKVGIKSNLAIIDGDKVIEMKPSDVDKGIAARKICDLYQP--------
S_cer_TSL1   934 --ENAEDQDRVASVIGDAITHINTVFDHRGIHAYVYKNVVSVQQVGLSLSAAQFLFRFYNSA--------
S_cer_TPS3   899 --ENAEDQDRVPTVIGEAITHINTLFDDRDIHAYVHKDIVFVQQTGLALAAAEFLMKFYNSG--------
S_cer_TPS2   696 --RRTVPELG-EFHAKELKEKLLSFTDDFDLEVMDGKANIEVRPRFVNKGEIVKRLVWHQHGKPQDMLKG
S_lep_TPSP   734 --KYADVEFGRVQARDMLQHLWTGPISNAAVDVVQGGKSVEVRPVGVSKGSAIDRILGEIVH--------
E_coli_TPP   134 --RQAPQ--HEDALMTLAQRITQ---IWPQMALQQGKCVVEIKPRGTSKGEAIAAFMQEAPFIG------
T_acid_TPP   136 GLMGADMKPKLRSRIEEIARIF-------GVETYYGKMIIELRVPGVNKGSAIRSVRGERP---------

                     Motif III

T_ten_TPSP   677 -----------DFVFVAGDDET-DEGMFEVAPQ-SAYTVKVGPGPTLAKFRVGDYRGLRSLLEQLRPP*
C_hut_TPSP   681 -----------DFILSIGDDRT-DEDMFKALPD-NALTIKVGVKNSSAKFTIKTQEEVMTILNTFL*
S_cer_TSL1   994 -----------SDPLDTSSGQI-TNIQTPSQQN-PSDQEQQPPASPTVSMNHIDFACVSGSSSPVLEPLF
S_cer_TPS3   959 -----------VSPTDNS--RI-SLSRTSSSMS-VGNNKKH-------FQNQVDFVCVSGSTSPIIEPLF
S_cer_TPS2   763 ISEKLPKDEMPDFVLCLGDDFT-DEDMFRQLNT-IETCWKEKYPDQKNQWGNYGFYPVTVGSASKKTVAK
S_lep_TPSP   794 ---SKHMTIPIDYVLCIGHFLSKDEDIYTFFEP-ELPLLDRDSSTSNGGKPLGGKLPIDRKSSKSSSRMK
E_coli_TPP   191 -----------RTPVFLGDDLT-DESGFAVVNRLGGMSVKIGTGATQASWRLAGVPDVWSWLEMITTALQ
T_acid_TPP   190 ------------AIIAGDDAT-DEAAFE-AND-DALTIKVGEGETHAKFHVADYIEMRKILKFIEMLGV

T_ten_TPSP                                            Stop  731 aa
C_hut_TPSP                                            Stop  733 aa
S_cer_TPS1                                            Stop  474 aa
S_cer_TSL1 1051 KLVNDEASEGQVKAGHAI 1068 --- *Stop 1098 aa
S_cer_TPS3 1007 KLVKQEVEKNNLKFGYTI 1024 --- *Stop 1054 aa
S_cer_TPS2  831 AHLTDPQQVLETLGLLVG  848 --- *Stop  896 aa
S_lep_TPSP  860 PPVSSPKSPGRGSEQQQQ  877 --- *Stop  994 aa
E_coli_TPS                                            Stop  474 aa
T_acid_TPS                                            Stop  441 aa
E_coli_TPP  249 QKRENNRSDDYESFSRSI*                       Stop  266 aa
T_acid_TPP  244 QKKQ*                                     Stop  247 aa
```

Figure S5: Sequence alignment of fused TPSP as well as single domain TPS and TPP proteins. Abbreviations and GeneBank accession numbers: *Thermoproteus tenax* (T_ten_TPSP, CCC81939), *Cytophaga hutchinsonii* (C_hut_TPSP, ABG57690), *Saccharomyces cerevisiae* (S_cer_TPS1 (AAT93166), S_cer_TPS2 (EDV08225), S_cer_TSL1 (CAY81720) and S_cer_TPS3 (EEU06667)), *Selaginella lepidophylla* (S_lep_TPSP, AAD00829)), *Escherichia coli* (E_coli_TPS, AAC74966) and *Thermoplasma*

acidophilum (T_acid_TPP, CAC12334). The alignment was constructed using Clustal W 1.83 (14) and manual refinement was done in the edit program of the MUST package (13). The amino acid residues involved in binding of G6P (▲) and UDPG (♦) in the TPS domains as well as those contributing to the active site in TPP domains (●) as deduced from the crystal structures of the *E. coli* TPS [7,8] and the *T. acidophilum* TPP [3] are indicated above the sequences. The three conserved motifs present in the TPP enzymes and other proteins of the HAD superfamily are shown in shaded boxes including the highly conserved motif residues highlighted in red [3]. Sequence stops are indicated by asterisks and the respective positions are given.

Figure S6

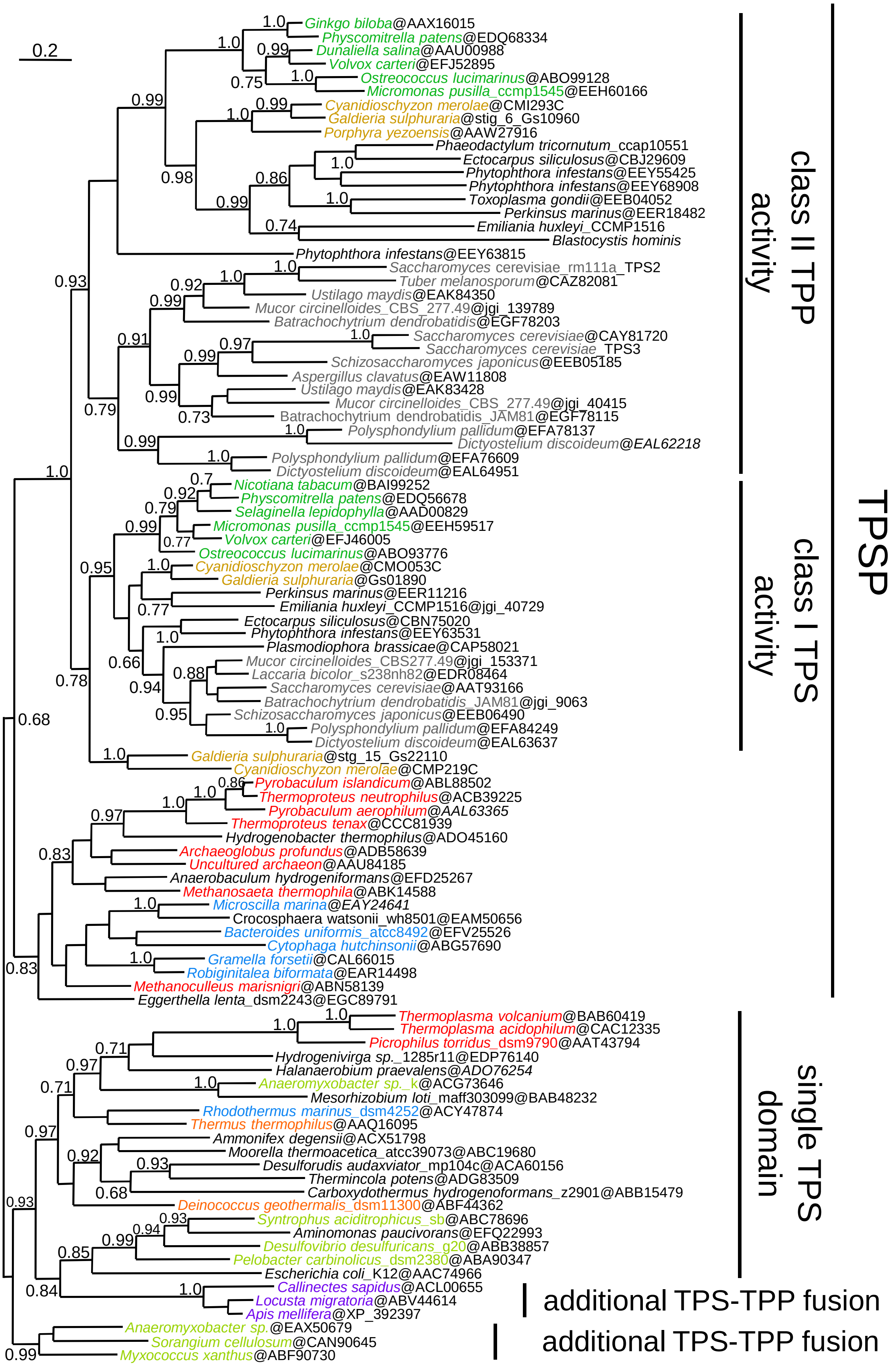

0.2
Ginkgo biloba@AAX16015
Physcomitrella patens@EDQ68334
Dunaliella salina@AAU00988
Volvox carteri@EFJ52895
Ostreococcus lucimarinus@ABO99128
Micromonas pusilla_ccmp1545@EEH60166
Cyanidioschyzon merolae@CMI293C
Galdieria sulphuraria@stig_6_Gs10960
Porphyra yezoensis@AAW27916
Phaeodactylum tricornutum_ccap10551
Ectocarpus siliculosus@CBJ29609
Phytophthora infestans@EEY55425
Phytophthora infestans@EEY68908
Toxoplasma gondii@EEB04052
Perkinsus marinus@EER18482
Emiliania huxleyi_CCMP1516
Blastocystis hominis
Phytophthora infestans@EEY63815
Saccharomyces cerevisiae_rm111a_TPS2
Tuber melanosporum@CAZ82081
Ustilago maydis@EAK84350
Mucor circinelloides_CBS_277.49@jgi_139789
Batrachochytrium dendrobatidis@EGF78203
Saccharomyces cerevisiae@CAY81720
Saccharomyces cerevisiae_TPS3
Schizosaccharomyces japonicus@EEB05185
Aspergillus clavatus@EAW11808
Ustilago maydis@EAK83428
Mucor circinelloides_CBS_277.49@jgi_40415
Batrachochytrium dendrobatidis_JAM81@EGF78115
Polysphondylium pallidum@EFA78137
Dictyostelium discoideum@EAL62218
Polysphondylium pallidum@EFA76609
Dictyostelium discoideum@EAL64951
Nicotiana tabacum@BAI99252
Physcomitrella patens@EDQ56678
Selaginella lepidophylla@AAD00829
Micromonas pusilla_ccmp1545@EEH59517
Volvox carteri@EFJ46005
Ostreococcus lucimarinus@ABO93776
Cyanidioschyzon merolae@CMO053C
Galdieria sulphuraria@Gs01890
Perkinsus marinus@EER11216
Emiliania huxleyi_CCMP1516@jgi_40729
Ectocarpus siliculosus@CBN75020
Phytophthora infestans@EEY63531
Plasmodiophora brassicae@CAP58021
Mucor circinelloides_CBS277.49@jgi_153371
Laccaria bicolor_s238nh82@EDR08464
Saccharomyces cerevisiae@AAT93166
Batrachochytrium dendrobatidis_JAM81@jgi_9063
Schizosaccharomyces japonicus@EEB06490
Polysphondylium pallidum@EFA84249
Dictyostelium discoideum@EAL63637
Galdieria sulphuraria@stg_15_Gs22110
Cyanidioschyzon merolae@CMP219C
Pyrobaculum islandicum@ABL88502
Thermoproteus neutrophilus@ACB39225
Pyrobaculum aerophilum@AAL63365
Thermoproteus tenax@CCC81939
Hydrogenobacter thermophilus@ADO45160
Archaeoglobus profundus@ADB58639
Uncultured archaeon@AAU84185
Anaerobaculum hydrogeniformans@EFD25267
Methanosaeta thermophila@ABK14588
Microscilla marina@EAY24641
Crocosphaera watsonii_wh8501@EAM50656
Bacteroides uniformis_atcc8492@EFV25526
Cytophaga hutchinsonii@ABG57690
Gramella forsetii@CAL66015
Robiginitalea biformata@EAR14498
Methanoculleus marisnigri@ABN58139
Eggerthella lenta_dsm2243@EGC89791
Thermoplasma volcanium@BAB60419
Thermoplasma acidophilum@CAC12335
Picrophilus torridus_dsm9790@AAT43794
Hydrogenivirga sp._1285r11@EDP76140
Halanaerobium praevalens@ADO76254
Anaeromyxobacter sp._k@ACG73646
Mesorhizobium loti_maff303099@BAB48232
Rhodothermus marinus_dsm4252@ACY47874
Thermus thermophilus@AAQ16095
Ammonifex degensii@ACX51798
Moorella thermoacetica_atcc39073@ABC19680
Desulforudis audaxviator_mp104c@ACA60156
Thermincola potens@ADG83509
Carboxydothermus hydrogenoformans_z2901@ABB15479
Deinococcus geothermalis_dsm11300@ABF44362
Syntrophus aciditrophicus_sb@ABC78696
Aminomonas paucivorans@EFQ22993
Desulfovibrio desulfuricans_g20@ABB38857
Pelobacter carbinolicus_dsm2380@ABA90347
Escherichia coli_K12@AAC74966
Callinectes sapidus@ACL00655
Locusta migratoria@ABV44614
Apis mellifera@XP_392397
Anaeromyxobacter sp.@EAX50679
Sorangium cellulosum@CAN90645
Myxococcus xanthus@ABF90730
class II TPP activity
class I TPS activity
TPSP
single TPS domain
additional TPS-TPP fusion
additional TPS-TPP fusion

Figure S6: Phylogenetic analysis of the TPS domain of fused TPSP and single domain TPS proteins with Bayesian inference

The same dataset as in Figure 5 was analyzed with Bayesian Inference using the program PhyloBayes with the site-heterogeneous empirical profile mixture CATfixC20+Γ4 model. Posterior predictive (PP) values ≥ 0.65 are given at internal branches. The following groups were colored: unikonts (grey); red algae (brown), green plants (green); Archaea (red); Bacteroidetes (blue); Delta-Proteobacteria (lemon); Deinococci-Thermus (orange) and Pan-Crustacean (magenta). The scale bar indicates the mean number of inferred substitutions per site.

Figure S7a

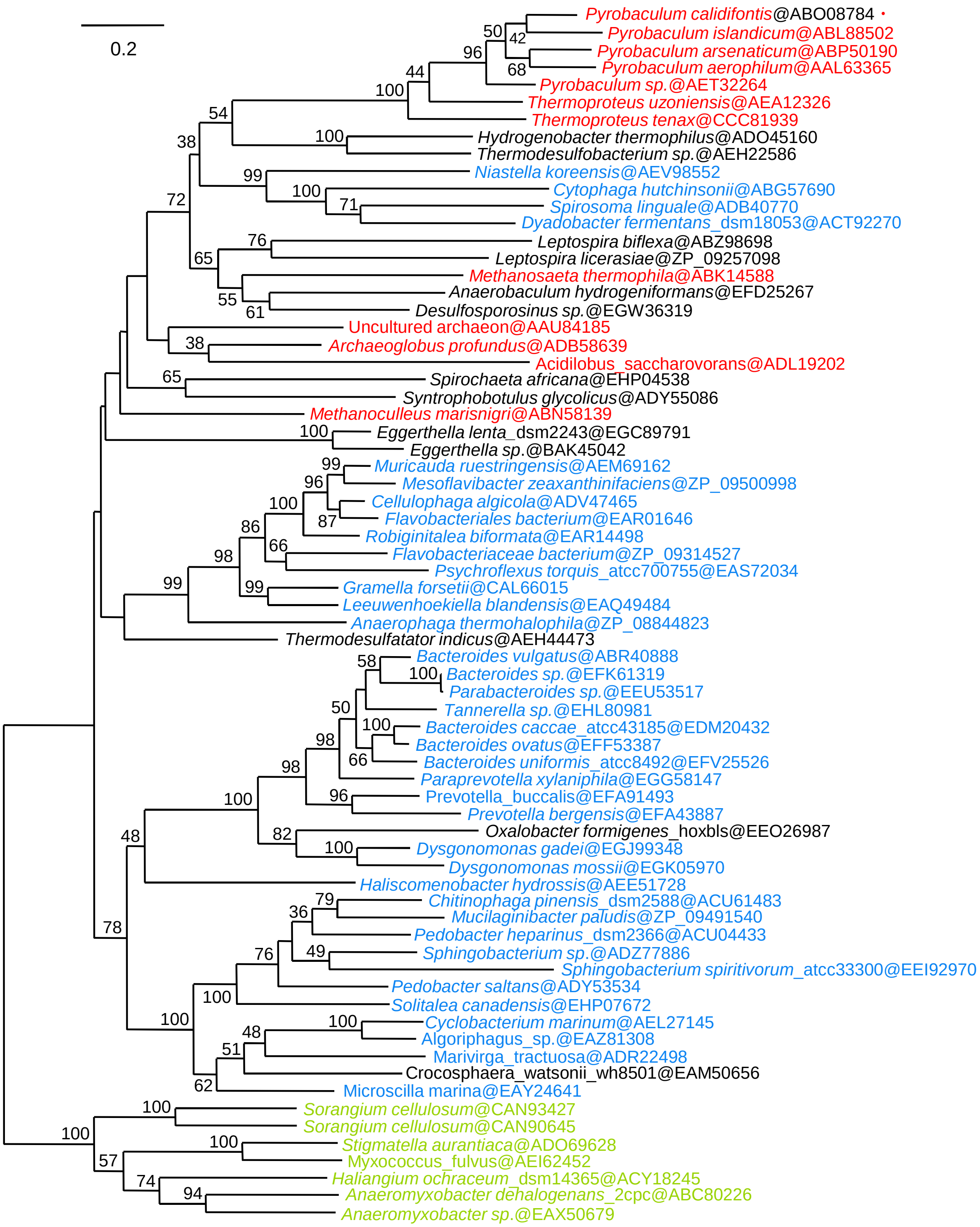

Figure S7a: Detailed ML phylogenetic analysis of the fused prokaryotic TPSP sequences.

The phylogenetic tree was inferred by RAxML with a LG+F+Γ4 model [9] of sequence evolution based on 70 sequences and 467 positions. The tree was rooted with deltaproteobacterial sequences (Myxococcales), which most likely represent an independent TPS+TPP fusion event. Bootstrap (BS) values are only indicated at internal branches if they are 30% or higher. The same color code is used as in Figure S6. The scale bar indicates the mean number of inferred substitutions per site.

Figure S7b

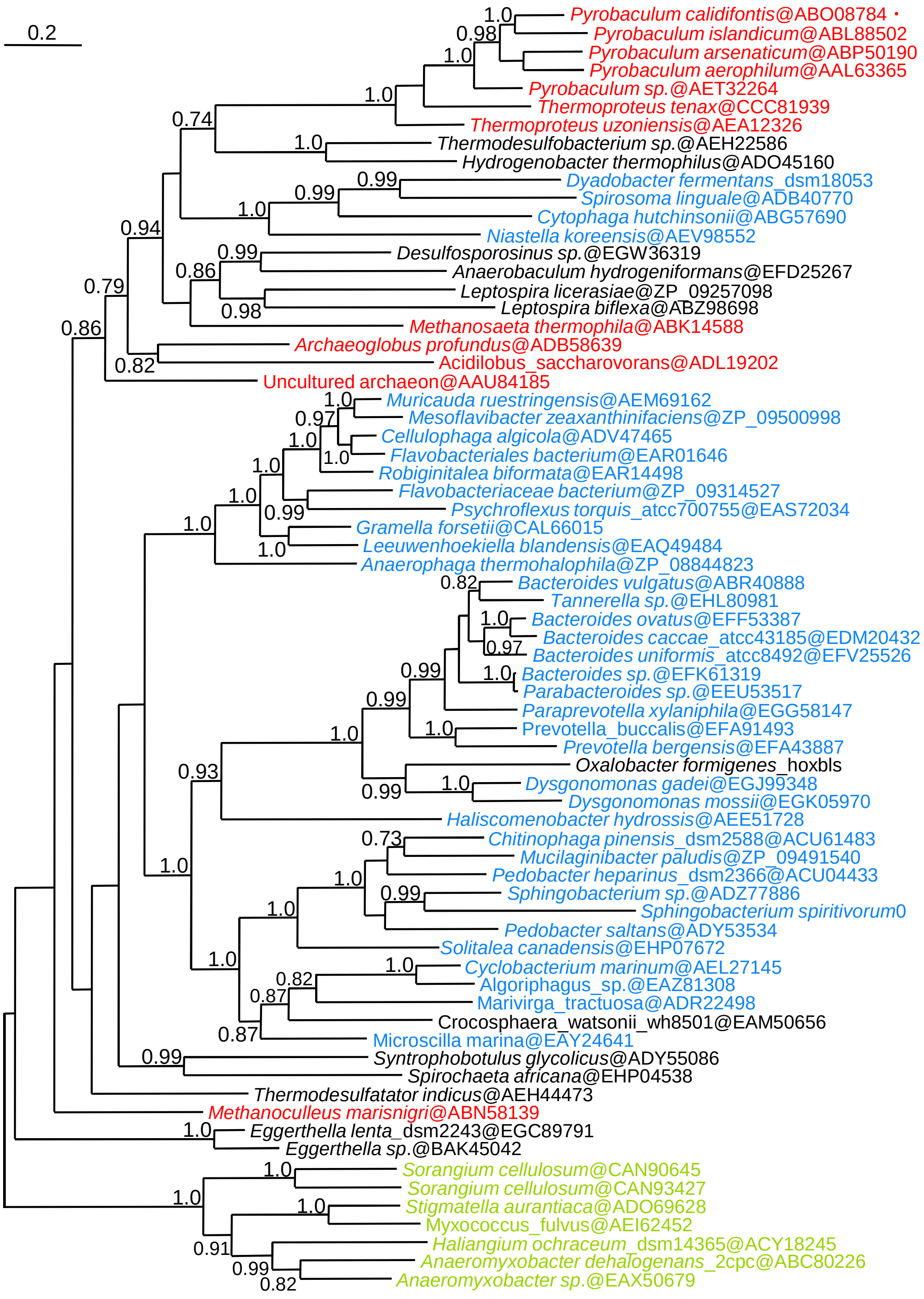

Figure S7b: Detailed phylogenetic analysis of the fused prokaryotic TPSP sequences using Bayesian inference.
The same dataset as in Figure S7a was analyzed with Bayesian Inference using the program PhyloBayes with the site-heterogeneous empirical profile mixture CATfixC60+Γ4 model. The same cut-off score and color coding was applied as in Figure S6. The scale bar indicates the mean number of inferred substitutions per site.

Figure S8a

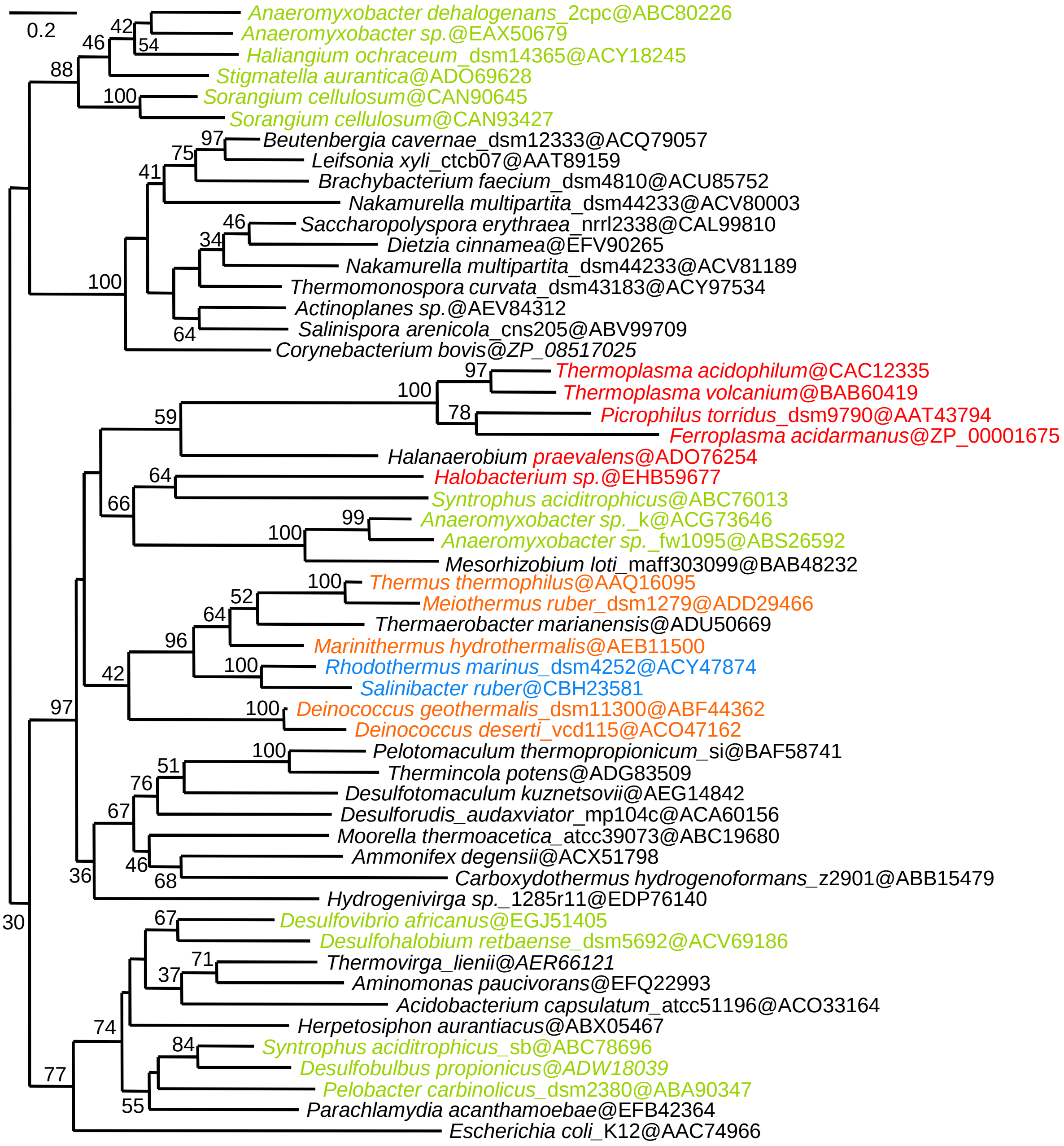

Figure S8a: ML phylogenetic analysis focused on prokaryotic single TPS domain sequences.

Due to their large number most of the non-fused prokaryotic TPS sequences were not incorporated into the phylogenetic tree. The vast majority of these sequences are from Bacteria, with only a few Archaea (red) rather randomly distributed suggesting that their sequences originated in several independent HGT events with Bacteria. There are no TPS sequences in Chlorobi, the sistergroup of Bacteroidetes, and also in other bacterial groups including Thermotogales, Spriochaetes, Epsilon-Proteobacteria and Fusobacteria, no TPS enzymes were identified. In other groups the number of species with TPS sequences is more or less reduced, within cyanobacteria to about one third, within the Firmicutes, Bacilli are devoid of them, and they are found in only twelve out of more than 200 Clostridia. In contrast, Actinobacteria are found in two large clusters and there is even more than one

sequence in certain genomes. The phylogenetic tree was inferred by RAxML with a LG+F+Γ4 model of sequence evolution based on 54 sequences and 282 positions [9]. The analysis shows the relationship between the only known non-fused single TPS domain enzyme in Bacteroidetes, i.e. those of *Rhodothermus* and *Salinibacter* (blue), whose phylogenetic position is basal as the sistergroup of all other Bacteroidetes, and the Thermales as part of the Deinococcus-Thermus phylum (orange). The high BS value of 96% that unites the two Bacteroidetes sequences with the Thermales argues together with the other facts strongly in favor of an HGT event early in the evolution of the phylum Bacteroidetes. The tree was also rooted with deltaproteobacterial sequences (Myxococcales). The cut-off score and color coding is the same as used in Figure S6. The scale bar indicates the mean number of inferred substitutions per site.

Figure S8b

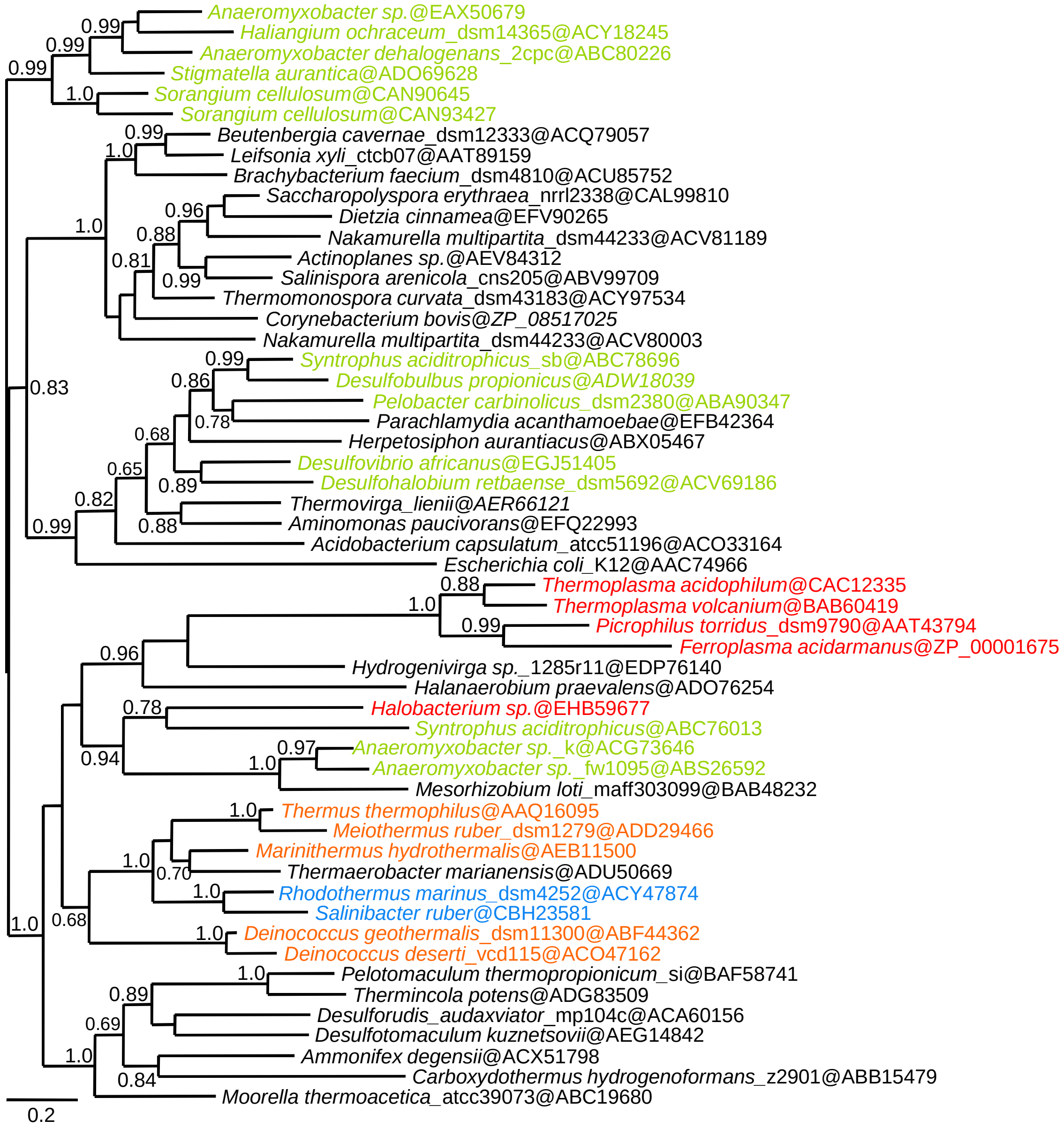

Figure S8b: Phylogenetic analysis focused on prokaryotic single TPS domain sequences using Bayesian inference.

The same dataset as in Figure S8a was analyzed with Bayesian Inference using the program PhyloBayes with the site-heterogeneous empirical profile mixture CATfixC40+Γ4 model. The same cut-off score and color coding was applied as in Figure S6. The scale bar indicates the mean number of inferred substitutions per site.

SUPPLEMENTARY REFERENCES

1. Kouril T, Zaparty M, Marrero J, Brinkmann H, Siebers B (2008) A novel trehalose synthesizing pathway in the hyperthermophilic Crenarchaeon *Thermoproteus tenax*: the unidirectional TreT pathway. Arch Microbiol 190: 355-369.
2. Chen YS, Lee GC, Shaw JF (2006) Gene cloning, expression, and biochemical characterization of a recombinant trehalose synthase from *Picrophilus torridus* in Escherichia coli. J Agric Food Chem 54: 7098-7104.
3. Rao KN, Kumaran D, Seetharaman J, Bonanno JB, Burley SK, et al. (2006) Crystal structure of trehalose-6-phosphate phosphatase-related protein: biochemical and biological implications. Protein Sci 15: 1735-1744.
4. Maruta K, Mitsuzumi H, Nakada T, Kubota M, Chaen H, et al. (1996) Cloning and sequencing of a cluster of genes encoding novel enzymes of trehalose biosynthesis from thermophilic archaebacterium *Sulfolobus acidocaldarius*. Biochim Biophys Acta 1291: 177-181.
5. Qu Q, Lee SJ, Boos W (2004) TreT, a novel trehalose glycosyltransferring synthase of the hyperthermophilic archaeon *Thermococcus litoralis*. J Biol Chem 279: 47890-47897.
6. Ryu SI, Park CS, Cha J, Woo EJ, Lee SB (2005) A novel trehalose-synthesizing glycosyltransferase from *Pyrococcus horikoshii*: molecular cloning and characterization. Biochem Biophys Res Commun 329: 429-436.
7. Gibson RP, Tarling CA, Roberts S, Withers SG, Davies GJ (2004) The donor subsite of trehalose-6-phosphate synthase: binary complexes with UDP-glucose and UDP-2-deoxy-2-fluoro-glucose at 2 A resolution. J Biol Chem 279: 1950-1955.
8. Gibson RP, Turkenburg JP, Charnock SJ, Lloyd R, Davies GJ (2002) Insights into trehalose synthesis provided by the structure of the retaining glucosyltransferase OtsA. Chem Biol 9: 1337-1346.
9. Stamatakis A, Hoover P, Rougemont J (2008) A rapid bootstrap algorithm for the RAxML Web servers. Syst Biol 57: 758-771.
